# Chromatin unwinding state for in situ identification and lineage tracing of circulating tumor cells

**DOI:** 10.1002/imt2.70119

**Published:** 2026-03-09

**Authors:** Bin Ye, Zhen Wang, Yinqi Bai, Xu Zhang, Rui Zhang, Jingru Lian, Xuefei Liu, Yan Zhang, Zhiyuan Xu, Li Yang, Haiman Jin, Fang Chen, Zhihao Xie, Ping Zhou, Jun Tan, Shan Zeng, Changzheng Du, Yang Min, Xiaomin Ni, Jingxian Duan, Zhicheng Li, Hui Yang, Yunpeng Cai, Hongyan Wu, Catherine C. Liu, Jing Cai, Yi Lu, Jian Zhang, Hao Wu, Hairong Zheng, Longqi Liu, Xin Hong, Hao Yu

**Affiliations:** ^1^ Institute of Biomedical and Health Engineering, Shenzhen Institute of Advanced Technology Chinese Academy of Sciences Shenzhen China; ^2^ School of Life Sciences and Medical Engineering Anhui University Hefei China; ^3^ Department of Biochemistry, SUSTech Homeostatic Medicine Institute, School of Medicine Southern University of Science and Technology Shenzhen China; ^4^ BGI‐Research Hangzhou China; ^5^ School of Mathematical Sciences South China Normal University Guangzhou China; ^6^ Research Center for Biomedical Information Technology, Shenzhen Institute of Advanced Technology Chinese Academy of Sciences Shenzhen China; ^7^ Department of Clinical Oncology University of Hong Kong‐Shenzhen Hospital Shenzhen China; ^8^ Department of Radiotherapy The First Affiliated Hospital of Hainan Medical University Haikou China; ^9^ Department of Neurosurgery, Xiangya Hospital Central South University Changsha China; ^10^ Department of Oncology, Xiangya Hospital Central South University Changsha China; ^11^ Department of Applied Mathematics The Hong Kong Polytechnic University Hong Kong China; ^12^ Department of Health Technology and Informatics The Hong Kong Polytechnic University Hong Kong China; ^13^ Joint Laboratory of Guangdong‐Hong Kong Universities for Vascular Homeostasis and Diseases, SUSTech Homeostatic Medicine Institute, School of Medicine Southern University of Science and Technology Shenzhen China; ^14^ Department of Human Cell Biology and Genetics, School of Medicine Southern University of Science and Technology Shenzhen China; ^15^ Clinical Research Center the First People's Hospital of Foshan (The Affiliated Foshan Hospital of Southern University of Science and Technology), School of Medicine, Southern University of Science and Technology Shenzhen China; ^16^ Faculty of Computer Science and Control Engineering, Shenzhen Institute of Advanced Technology Chinese Academy of Sciences Shenzhen China; ^17^ The Key Laboratory of Biomedical Imaging Science and System Chinese Academy of Sciences Shenzhen China; ^18^ Key University Laboratory of Metabolism and Health of Guangdong Southern University of Science and Technology Shenzhen China; ^19^ Guangdong Provincial Key Laboratory of Cell Microenvironment and Disease Research Southern University of Science and Technology Shenzhen China; ^20^ Department of Biomedical Engineering Shenzhen University of Advanced Technology Shenzhen China

## Abstract

The detection of circulating tumor cells (CTCs) through liquid biopsy offers a non‐invasive approach for accurately monitoring cancer dissemination and evaluating therapeutic efficiency. However, their rarity and heterogeneity limit conventional tumor antigen labelling‐based methods in identifying and tracing CTCs. Here, we developed a novel metric, termed chromatin unwinding state (CUS), which leverages activated transcriptional regions related to cell‐identity processes from single‐cell transcriptomic data while overcoming technical variances. Using CUS features, we trained attention‐based neural network models, panCTC, to in situ identify and lineage trace rare single CTCs directly from 5 mL of peripheral blood mononuclear cells scRNA‐seq without enrichment. We benchmarked panCTC on various in silico‐simulated, public, and in‐house sequenced data, demonstrating its robustness across sample types and platforms. PanCTC could provide real‐time scRNA‐seq profiles of fresh CTCs, supporting early cancer detection and targeted anti‐metastatic therapy.

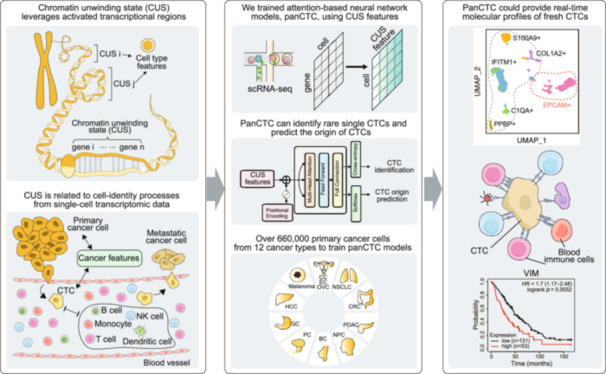

## CONFLICT OF INTEREST STATEMENT

The authors declare no conflicts of interest.

## ETHICS STATEMENT

All participants provided written informed consent, and the research has been approved by the Medical Ethics Committee of the University of Hong Kong‐Shenzhen Hospital (No. [2019]049‐001) and the First Affiliated Hospital of Hainan Medical University (No. [2021]126).

To the Editor,

Circulating tumor cells (CTCs) are key metastatic precursors, yet their extreme rarity and molecular heterogeneity—including downregulation of epithelial markers (e.g., *EpCAM*⁻/*CK*⁻) [1], acquisition of hybrid phenotypes (e.g., *EpCAM*⁺*CD68*⁺) [2], or taking up platelets [3]—severely challenge their detection. Current marker‐dependent platforms (e.g., CellSearch® or CTC‐iChip) [4, 5] often miss these aggressive CTC subsets, compromising their clinical translation.

Current single‐cell analysis tools, including general‐purpose annotation tools (e.g., scATOMIC [6] and CopyKAT [7]) and large‐scale pretrained foundation models (e.g., scGPT [8] and scFoundation [9]), are not designed for CTC identification. Although dedicated tools such as CTCTracer [10] and iCTC [11] have been developed, they are trained on limited CTC datasets and exhibit poor predictive power. The marked phenotypic plasticity of CTCs further complicates the reliable determination of their cellular origin. Therefore, the core challenge in accurately identifying and tracing CTCs from scRNA‐seq data lies in the ability to extract both pan‐cancer conserved features and disease‐specific heterogeneous signatures from tumor transcriptomic data.

To address these limitations, we introduced the concept of “chromatin unwinding state” (CUS) derived from scRNA‐seq data. In a given cell, certain chromatin regions adopt an unwound architecture, creating a permissive nanoenvironment that facilitates transcriptional activity by modulating molecular accessibility and reaction energetics [12]. These chromatin regions span sub‐megabase domains within chromosomal territories [13]. CUS captures transcriptional activation patterns linked to cell identity and biological state, which we hypothesized are conserved from primary tumors to CTCs and can serve as robust, marker‐independent signatures.

Here, we present panCTC, a deep‐learning algorithm that extracts pan‐cancer conserved CUS features to identify and trace CTCs directly from scRNA‐seq data of peripheral blood mononuclear cells (PBMCs), without prior enrichment or labeling. PanCTC detects classical and non‐classical CTCs at single‐cell resolution, offering a powerful new approach to advance liquid biopsy and precision oncology.

## THE PANCTC FRAMEWORK FOR IDENTIFICATION AND LINEAGE TRACING OF CTCS USING PRIMARY TUMOR CUS FEATURES

We propose the CUS as a cell‐intrinsic metric for quantifying local transcriptional activity and present the panCTC framework. This framework uses CUS features derived from primary tumors to identify CTCs and trace their tissue of origin.

**FIGURE 1:**
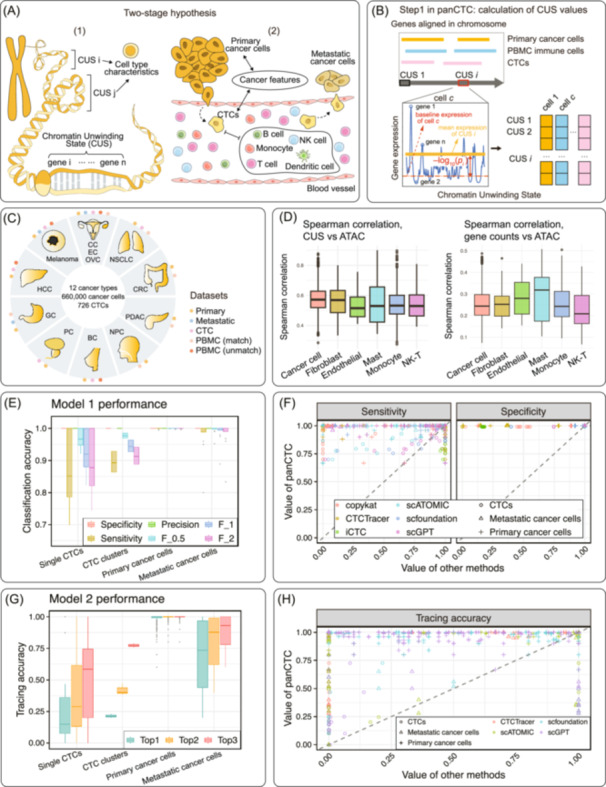
Overview of the panCTC framework, underlying hypotheses, deep learning algorithm, dataset composition, and performance in identifying CTCs and tracing their lineage. (A) Two sequential hypotheses underpin panCTC: (1) chromatin unwinding state (CUS) profiles reflect lineage‐inherited transcriptional activity states, and (2) CTCs retain CUS features of their corresponding primary tumors. (B) Schematic of the CUS algorithm. For the entire overview and schematic of panCTC please refer to Text S1 and Figure S1. (C) Datasets used in this study, including public and newly sequenced data from primary tumors (12 cancer types), metastatic tumors, PBMCs from tumor patients and healthy donors, immune cells, and experimentally confirmed CTCs (e.g., via CellSearch). Details are provided in Methods and Tables S1–S5. (D) At the single‐cell resolution, CUS showed substantially stronger correlation with ATAC‐seq peaks than gene expression across 6 common cell types. (E) Performance of panCTC Model 1 in identifying CTCs, CTC‐clusters, primary cancer cells, and metastatic cancer cells across 1136 unseen datasets. Details are shown in Figure S5B–D. (F) Comparison of panCTC with existing methods on the 102 unseen pseudo‐PBMC test sets. Points above the diagonal indicate superior performance of panCTC (*y*‐axis) over current methods (x‐axis). Detailed results are shown in Figure S5F–H. (G) Top tracing accuracy of panCTC Model 2 for assigning cancer types to CTCs, CTC‐clusters, primary cancer cells, and metastatic cancer cells. Details are shown in Figure S6B–F. (H) Comparison of cancer‐type tracing performance. Points above the diagonal denote test sets where panCTC (*y*‐axis) outperforms current methods (*x*‐axis). Details are shown in Figure S6G–I.

We hypothesize that (1) CUS profiles reflect lineage‐inherited transcriptional activity states, and (2) CTCs retain CUS features of their corresponding primary tumors (Figure 1A). Accordingly, the panCTC framework first converts the single‐cell unique molecular identifier (UMI) count matrix into a CUS matrix. The CUS value is computed by statistically comparing the expression level of a genomic window (e.g., spanning 10 consecutive genes) against a cell‐specific expression baseline, without relying on population‐level references (Figure 1B, Text S1, Figure S1A). Subsequently, an attention‐based network performs a two‐tier classification: first distinguishing CTCs from other cells, then classifying CTCs into one of 12 candidate primary tumor types (Text S1, Figure S1B,C).

For model training and validation, we integrated over 660,000 cells from 42 datasets, including primary and metastatic tumor cells from 12 cancer types, PBMCs from healthy donors and cancer patients, and previously published CTC scRNA‐seq data (Figure 1C, Tables S1–S5).

## CUS: A TRANSCRIPTOMIC PROXY FOR CHROMATIN ACCESSIBILITY CAPTURING LINEAGE AND MALIGNANCY OF CTCS ACROSS CANCERS

To validate the biological relevance of CUS, we first assessed transcriptomic profiles via CUS and measured its correlation with chromatin accessibility (ATAC‐seq peaks) (Figure S2A,B). Across six major cell types, CUS showed a substantially stronger association with promoter/enhancer ATAC peaks than gene expression alone (Figure 1D). In chromatin regions where CUS and ATAC peaks were highly correlated in kidney cancer cells, we identified pro‐tumor transcription factors such as *BACH1* and *ZNF12*, which are not readily discernible from expression data alone (Figure S2C). As such, CUS serves as a transcriptomic proxy for chromatin accessibility.

CUS also effectively captured cell‐type‐specific signatures. Canonical gene markers were enriched within corresponding CUS regions (e.g., the immune‐cell‐associated CUS‐255 region contains *CD45*, and the epithelial‐cancer‐associated CUS‐373 region contains *EPCAM*) (Figure S3A). Gene sets derived from immune‐ and tumor‐specific CUS were enriched for cell type‐specific functional pathways, respectively (Figure S3B, Tables S6–S7).

Moreover, CUS preserved cell identity while minimizing technical noise across scRNA‐seq platforms (10X Genomics, Smart‐seq, DNBelab), outperforming raw UMI counts in terms of data consistency across platforms (*p* < 0.001, Kruskal–Wallis test) (Figure S4). These findings demonstrate that high‐CUS regions are associated with multi‐gene chromosomal contexts correlated with chromatin states, regulatory factors, and cellular functions, providing a stable new metric for cell identification.

## PANCTC ENABLES ROBUST CTCS IDENTIFICATION AND LINEAGE TRACING

PanCTC comprises two AI models trained on CUS matrices from scRNA‐seq data of primary tumor tissues. Both models were subsequently validated using computationally generated pseudo‐PBMC datasets containing unseen cancer cells, including previously confirmed CTCs by established microfluidic isolation methods, primary and metastatic tumor cells, all with ground‐truth annotations (Figure S5A).

PanCTC's Model 1, for CTCs identification, achieved exceptional performance (sensitivity: 0.962 ± 0.079; specificity: 1.0 ± 0) across 1136 unseen datasets (Figure 1E, Figure S5B–D). It surpassed six state‐of‐the‐art methods (scATOMIC [6], CopyKAT [7], scGPT [8], scFoundation [9], CTCTracer [10], and iCTC [11]) in accuracy (Figure 1F, Figure S5F–H) and maintained the lowest false‐positive rate of 0.001 ± 0.001 in 21 healthy‐donor PBMC samples (Figure S5E).

PanCTC's Model 2, for twelve‐class cancer classification, far exceeded the random baseline (0.083) on 260 independent datasets, achieving near‐perfect accuracy on primary cancer cells (1 ± 0.002), high accuracy on metastatic cells (0.923 ± 0.067), and moderate but highly variable accuracy on experimentally isolated CTCs (0.529 ± 0.335) (Figure 1G, Figure S6A–F). It demonstrated significantly superior prediction accuracy compared to existing methods (scATOMIC [6], scGPT [8], scFoundation [9], and CTCTracer [10]) (Figure 1H, Figure S6G–I).

**FIGURE 2:**
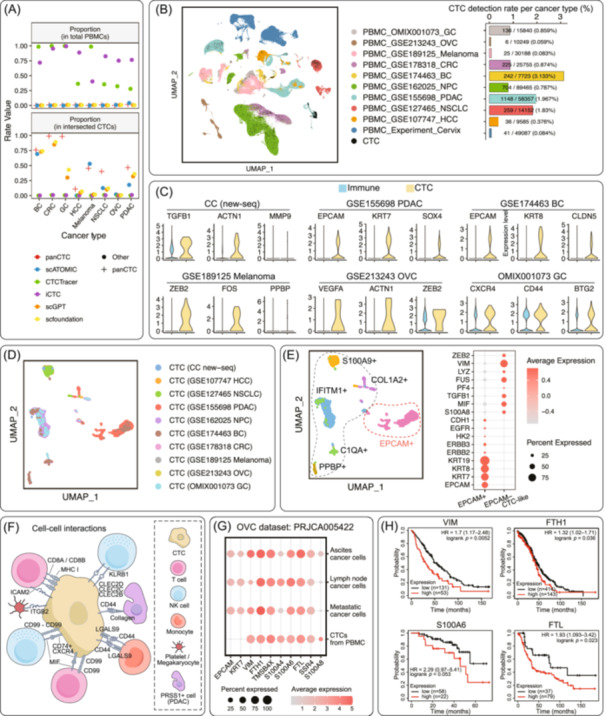
Overview of the performance evaluation of the panCTC method in identifying circulating tumor cells (CTCs), encompassing their molecular and subtype characteristics, interactions with the immune microenvironment, and clinical prognostic relevance. (A) Dot plots of the proportion of CTCs identified by panCTC and six state‐of‐the‐art methods on unseen scRNA‐seq data of PBMCs across eight cancer types. The upper panel shows that panCTC identified the lowest proportion of CTCs (0.63%) among total PBMCs, consistent with prior knowledge that CTCs are extremely rare. The lower panel represents that the proportion of reliable CTCs, identified as those identified by at least three methods, among all CTCs identified by each method was the highest for panCTC (59.5%). (B) UMAP visualization of all cells from the analyzed PBMC samples (including newly sequenced samples from colorectal cancer (CC) patients). Cells identified as CTCs by panCTC are shown in black color (left panel). Barplot of the detection rate (CTCs per total cells) for each cancer type, ranging from 0.059% (ovarian cancer, OVC) to 3.133% (breast cancer, BC); CTC counts ranging from 6 (OVC) to 1148 (pancreatic ductal adenocarcinoma, PDAC) (right panel). At the sample level, detection rates ranged from 0.102% to 1.449%. panCTC achieved 100% patient‐level sensitivity (≥1 CTC/patient). (C) Expression levels of selected genes associated with prior CTC characteristics were higher in CTCs than in immune cells. (D) UMAP visualization of CTCs identified by panCTC across clinical PBMC datasets from patients across 10 cancer types. (E) All CTCs were divided into two groups based on epithelial gene markers (*EPCAM*, *KRT7*, *KRT8*, and *KRT19*): *EPCAM* ^+^ CTC and *EPCAM* ^–^ CTC‐like subpopulations. *EPCAM* ^–^ CTC‐like cells exhibited high expression of mesenchymal‐related gene markers such as *VIM*, *FUS*, and *TGFB1*. *EPCAM* ^–^ CTC‐like cells were further grouped into five subclusters, with highly expressed markers for each subcluster labeled in the UMAP plot (left panel). Differentially expressed genes between *EPCAM* ^+^ CTCs and *EPCAM* ^–^ CTC‐like cells were shown (right panel). (F) The main cell–cell interactions between CTCs and immune cells in PBMCs. Further details of the ligand–receptor pairs are provided in Figure S12. (G) Highly expressed markers shared by CTCs and metastatic cancer cells were derived from paired metastatic tumors, lymph nodes, and ascites. These CTCs were classified as *EPCAM* ^–^ type and shared common genes with metastatic cancer cells while also exhibiting distinct gene expression patterns. (H) Survival analyses of ovarian cancer patients (PRJCA005422) from both the TCGA and other GEO databases revealed that marker genes associated with CTCs identified by panCTC were significantly correlated with poor overall survival. Associations of the other four CTC‐related genes with CTCs with overall survival are shown in Figure S13G.

Finally, on unseen scRNA‐seq data of real PBMCs from eight cancer types, panCTC was benchmarked against high‐confidence CTCs. Given the distinct strengths of various existing deep learning methods for CTC identification and the lack of an established gold standard, we defined a set of high‐confidence CTCs derived from the intersection of at least three technical approaches. PanCTC exhibited the most stringent CTC detection rate (0.63%, panCTC‐identified CTCs in all PBMCs) and the highest overlap with high‐confidence CTCs (59.5%, high‐confidence CTCs in panCTC‐identified CTCs) (Figure 2A). It achieved patient‐level single‐cell detection rates 0.102%–1.449% (CTCs in PBMCs of one patient's blood sample) versus <0.5% (for current methods) and attained patient‐level sensitivity 100% versus 83.3%–97% (Figure 2B). These results establish panCTC as a novel platform leveraging conserved CUS features of primary tumor cells for CTCs detection and lineage tracing from millions of PBMCs.

## COPY NUMBER VARIATIONS AND SOMATIC MUTATIONS ANALYSES DEMONSTRATE MALIGNANCY OF POTENTIAL CTCS IDENTIFIED BY PANCTC

We further characterized CTCs identified by panCTC using copy number variations (CNVs) and somatic mutational analyses between CTCs and patient‐matched primary tumors. In pancreatic ductal adenocarcinoma (PDAC), CTCs exhibited common CNV patterns shared with specific tumor subclones (Figure S7). These shared CNVs involved genes related to the cytoskeleton, adhesion, and epithelial‐mesenchymal transition (e.g., *S100A8*, *VIM*, and *COL1A1*). In cervical cancer (CC), however, CNV analysis revealed only partial conservation at certain chromosomes between CTCs and primary tumors, likely due to evolutionary divergence between CTCs and primary tumor cells (Figure S8).

Subsequent analysis of somatic mutations in colorectal and breast cancer patients confirmed shared mutations in cancer‐related genes (including *CDKN2C*, *LMNA*, *MUC1*, *PDE4DIP*, *HRAS*, *B2M*, *COL1A1*, and *PIK3CA*) between CTCs and patient‐matched primary tumor cells (Figure S9). Together, these results indicate that CNV and mutation analyses support the malignancy nature of at least a fraction of CTCs identified by panCTC.

## PANCTC ENABLES SINGLE‐CELL PROFILING OF CTCS, REVEALING HETEROGENEOUS MOLECULAR COMPOSITION AND IMMUNE INTERACTIONS

PanCTC enables comprehensive single‐cell characterization of CTCs across 10 cancer types. Transcriptomic analysis revealed cancer‐type‐specific patterns: PDAC and breast cancer (BC) CTCs highly expressed epithelial (*EPCAM*, *KRT*s) and stemness (*SOX4*, *SOX9*) markers, while CTCs from cervical, lung, and gastric cancers displayed elevated proliferation (*DUSP1*, *BTG2*), migration (*S100A8*, *MMP9*, *VEGFA*), platelet interaction (*TUBB1*, *PPBP*), and oncogenesis (*JUN*, *FOS*) (Figure 2C, Figure S10A). EMT status varied, with PDAC/BC CTCs showing hybrid epithelial‐mesenchymal phenotypes and others being predominantly mesenchymal (Figure S10B–C).

CTCs were categorized into *EPCAM* ^+^ and *EPCAM* ^–^ subpopulations with distinct marker expression and functional profiles. Most *EPCAM* ^+^ CTCs were predominantly from PDAC and BC, whereas *EPCAM* ^–^ CTC‐like cells predominated in liver, lung, and other cancers, highly expressing non‐typical CTC markers (*C1QA*, *PPBP*) [1, 2, 3] (Figure 2D–E, Figure S11).

Ligand‐receptor analysis further elucidated potential mechanisms mediating CTC intravasation, survival, extravasation, and colonizing metastases. For instance, *ITGB*‐*ICAM* is involved in migration and CTC cluster formation [14], *MHC‐I* and *LGALS9*‐*CD44* may mediate immune evasion [15], *CLEC*‐*KLRB1* may protect CTCs from NK cell‐mediated lysis [16, 17], *CD99*‐*CD99* and *MIF*‐*CD74* can promote extravasation [18] (Figure 2F, Figure S12). Together, these findings demonstrate that panCTC can uncover the molecular heterogeneity and microenvironmental interactions of CTCs within the blood compartment.

## IN SITU PROFILING OF CTCS BY PANCTC UNCOVERS *EPCAM* ^–^ SUBSETS LINKED TO METASTASIS AND POOR PROGNOSIS

We further evaluated the clinical relevance of CTCs identified by panCTC. Analysis of PBMC scRNA‐seq data from NSCLC, BC, and GC patients with tumor stage revealed CTCs molecular features associated with disease progression. Metastatic patients exhibited significantly more CTCs (*n* = 525, detection rate 3.6%) than non‐metastatic patients (*n* = 111, detection rate 1.1%) (Figure S13A, Table S8).

CTCs were further subclassified into *EPCAM* ^+^ and *EPCAM* ^–^ subsets (Figure S13B). *EPCAM* ^+^ CTCs were mainly detected in metastatic BC patients, while *EPCAM* ^–^ CTC‐like cells were present across all patients (Figure S13C). Metastasis‐derived *EPCAM* ^–^ CTC‐like cells showed upregulation of adhesion, migration, and fusion markers (*S100A8*, *S100P*, *MMP9*) and enrichment in migration and potassium channel pathways (Figure S13D–E). Cell–cell communication analysis revealed *EPCAM* ^–^‐specific signals involving innate immunity (*GZMA*, *RETN*), platelet adhesion (*PF4*), and epithelial proliferation (*GRN*) (Figure S13F).

In an ovarian cancer (OVC) cohort [19], all panCTC‐detected CTCs were *EPCAM* ^–^ CTC‐like cells and shared expression of markers (e.g., *VIM* and *S100A4*) with metastatic cells from matched primary tumors, lymph nodes, and ascites (Figure 2G). High expression of these markers in bulk RNA datasets correlated with significantly reduced overall survival (Figure 2H, Figure S13G), supporting their potential as non‐invasive prognostic biomarkers. Therefore, panCTC enables molecular characterization of individual CTCs and may provide non‐invasive biomarkers with broad clinical implications.

## POTENTIAL APPLICATION AND LIMITATIONS

The in situ detection of CTCs within PBMC scRNA‐seq profiles offers considerable potential for cancer screening and metastasis research. In this study, we developed panCTC, an AI method leveraging CUS, genomic regions of high transcriptional activity inferred from scRNA‐seq, to accurately identify and trace CTCs.

PanCTC outperformed existing methods (scATOMIC [6], scGPT [8], CTCTracer [10], etc.) in challenging scenarios, such as samples with extremely low CTC counts or *EPCAM* ^–^ cases, or early‐stage disease. Its superior reliability stems from a design specialized for CTCs, utilizing robust CUS features and training expanded with millions of primary tumor cell data to overcome CTC‐sample limitations (Table S9).

Compared to current clinical liquid biopsy approaches (e.g., CellSearch [4] and ctDNA assays [20], Table S10), panCTC requires no physical enrichment and achieves higher single CTC detection sensitivity. It can trace CTC tissue origin using only peripheral blood, complementing imaging‐based diagnostics. Furthermore, panCTC enables functional characterization of CTCs, revealing insights into mechanisms like cluster formation, immune evasion [15], and extravasation [18] under native conditions.

Limitations include restricted cancer‐type coverage due to scRNA‐seq data availability, scarcity of annotated CTC datasets, and computational simplifications in CUS estimation. Expanding datasets, in vitro and animal models, and refining algorithms will enhance future performance.

In conclusion, panCTC provides a deep learning framework for the in situ identification and lineage tracing of CTCs directly from blood, independent of enrichment. It detects rare single CTCs and traces their origin using conserved CUS patterns, offering a powerful, minimally invasive platform for advancing metastasis research and liquid biopsy applications.

## AUTHOR CONTRIBUTIONS

**Bin Ye**: Conceptualization; methodology; data curation; software; investigation; writing—original draft; writing—review and editing; visualization. **Zhen Wang**: Methodology; software; data curation; investigation; writing—review and editing; visualization. **Yinqi Bai**: Methodology; software; data curation; investigation; writing—review and editing. **Xu Zhang**: Methodology; software; data curation; investigation; writing—review and editing. **Rui Zhang**: Methodology; software; data curation; investigation; writing—review and editing. **Jingru Lian**: Data curation; investigation; methodology; writing—review and editing; resources. **Xuefei Liu**: Methodology; data curation; investigation; writing—review and editing; resources. **Yan Zhang**: Investigation; formal analysis; data curation; writing—review and editing; resources. **Zhiyuan Xu**: Investigation; data curation; formal analysis; writing—review and editing; resources; funding acquisition. **Li Yang**: Investigation; formal analysis; data curation; writing—review and editing; resources. **Haiman Jin**: Investigation; writing—review and editing; formal analysis; data curation; resources. **Fang Chen**: Investigation; writing—review and editing; formal analysis; data curation; resources. **Zhihao Xie**: Formal analysis; investigation; data curation; resources; writing—review and editing. **Ping Zhou**: Investigation; writing—review and editing; formal analysis; data curation; resources; funding acquisition. **Jun Tan**: Investigation; writing—review and editing; formal analysis; data curation; resources. **Shan Zeng**: Investigation; writing—review and editing; formal analysis; data curation; resources. **Changzheng Du**: Investigation; writing—review and editing; formal analysis; data curation; resources. **Yang Min**: Investigation; formal analysis; data curation; writing—review and editing; resources. **Xiaomin Ni**: Data curation; resources; formal analysis; writing—review and editing; investigation. **Jingxian Duan**: Investigation; writing—review and editing; formal analysis; resources. **Zhicheng Li**: Investigation; writing—review and editing; formal analysis; resources. **Hui Yang**: Investigation; writing—review and editing; formal analysis; resources. **Yunpeng Cai**: Investigation; writing—review and editing; formal analysis; resources. **Hongyan Wu**: Investigation; writing—review and editing; formal analysis; resources. **Catherine C. Liu**: Investigation; writing—review and editing; formal analysis; resources. **Jing Cai**: Investigation; writing—review and editing; formal analysis; resources. **Yi Lu**: Funding acquisition; investigation; writing—review and editing; resources; formal analysis. **Jian Zhang**: Investigation; funding acquisition; writing—review and editing; formal analysis; resources. **Hao Wu**: Investigation; writing—review and editing; formal analysis; resources. **Hairong Zheng**: Resources; writing—review and editing. **Longqi Liu**: Writing—review and editing; resources. **Xin Hong**: Methodology; data curation; writing—review and editing; funding acquisition; investigation; resources; project administration; validation; supervision. **Hao Yu**: Project administration; resources; supervision; data curation; validation; writing—review and editing; writing—original draft; conceptualization; methodology; software; investigation; funding acquisition; visualization. All authors have read the final manuscript and approved it for publication.

## Supporting information


**Figure S1.** Overview of the panCTC framework for CTCs detection from scRNA‐seq data of PBMCs.
**Figure S2.** Correlation between scATAC‐seq chromatin accessibility and CUS values across cell types.
**Figure S3.** CUS values capture cell‐type‐specific transcriptional signatures.
**Figure S4.** CUS values enable cross‐platform integration while preserving cell‐type signatures.
**Figure S5.** Performance evaluation of panCTC Model 1 for CTC identification.
**Figure S6.** Performance evaluation of panCTC Model 2 for tracing the tissue origin of CTCs.
**Figure S7.** Subclustering of PDAC primary cancer cells and CNV similarity between CTCs and primary subclusters.
**Figure S8.** Subclustering of newly sequenced colorectal cancer (CC) samples and CNV similarity between CTCs and primary subclusters.
**Figure S9.** Somatic mutations shared between newly sequenced CTCs and matched primary cancer cells.
**Figure S10.** Biological characteristics of panCTC‐identified CTCs are consistent with established CTC knowledge.
**Figure S11.** Transcriptional profiles and pathway enrichment of *EPCAM* ^+^ vs. *EPCAM* ^–^ CTC‐like cells.
**Figure S12.** Cell–cell interactions between CTCs and immune cells in PBMCs.
**Figure S13.** Clinical relevance of in situ‐identified CTCs.

**Table S1.** Single‐cell RNA data of primary tumor and preprocessing parameters used in this study.
**Table S2.** Public scRNA data of 5 types of immune cells from healthy PBMC and preprocessing parameters used in this study.
**Table S3.** Single‐cell RNA data of CTCs and preprocessing parameters for them in this study.
**Table S4.** Public scRNA data of metastasis tumor and preprocessing parameters used in this study.
**Table S5.** New‐sequenced and public scRNA data of cancer patient PBMCs and healthy donor PBMCs, and their preprocessing parameters used in this study.
**Table S6.** Selected chromatin unwinding states (CUSs) of immune and tumor.
**Table S7.** Selected chromatin unwinding states (CUSs) of each cancer types.
**Table S8.** Tumor stage information of public patient PBMC samples selected to use in this study.
**Table S9.** A systematic comparison of panCTC versus existing deep learning techniques for CTC detection.
**Table S10.** A systematic comparison of panCTC versus with current clinical gold‐standard approaches.

## Data Availability

The data that support the findings of this study are available on request from the corresponding author. The data are not publicly available due to privacy or ethical restrictions. Due to ethical and legal restrictions, individual‐level data of patients cannot be made publicly available. Data are available from the corresponding author (hao.yu@siat.ac.cn) upon reasonable request and subject to local rules and regulations. This includes submitting a proposal to the management team, where upon approval, analysis needs to be done on a local server with protected access, complying with GDPR regulations. Code of panCTC is available at https://github.com/SiatBioInf/panCTC. Supplementary materials (methods, text, figures, tables, graphical abstract, slides, videos, Chinese translated version, and update materials) may be found in the online DOI or iMeta Science http://www.imeta.science/.
