# Supplementary material for: Chromatin unwinding state for in situ identification and lineage tracing of circulating tumor cells
Source: Imeta. 2026 Mar 9;5(2):e70119. doi: 10.1002/imt2.70119 (PMC13147939; doi:10.1002/imt2.70119)
Supplement: Supplementary file 1 — Figure S1. Overview of the panCTC framework for CTCs detection from scRNA‐seq data of PBMCs. Figure S2. Correlation between scATAC‐seq chromatin accessibility and CUS values across cell types. Figure S3. CUS values capture cell‐type‐specific transcriptional signatures. Figure S4. CUS values enable cross‐platform integration while preserving cell‐type signatures. Figure S5. Performance evaluation of panCTC Model 1 for CTC identification. Figure S6. Performance evaluation of panCTC Model 2 for tracing the tissue origin of CTCs. Figure S7. Subclustering of PDAC primary cancer cells and CNV similarity between CTCs and primary subclusters. Figure S8. Subclustering of newly sequenced colorectal cancer (CC) samples and CNV similarity between CTCs and primary subclusters. Figure S9. Somatic mutations shared between newly sequenced CTCs and matched primary cancer cells. Figure S10. Biological characteristics of panCTC‐identified CTCs are consistent with established CTC knowledge. Figure S11. Transcriptional profiles and pathway enrichment of EPCAM + vs. EPCAM – CTC‐like cells. Figure S12. Cell–cell interactions between CTCs and immune cells in PBMCs. Figure S13. Clinical relevance of in situ‐identified CTCs. [file IMT2-5-e70119-s001.docx]

**Supplementary information to**

**Chromatin unwinding state for in situ identification and lineage tracing of circulating tumor cells**

**Running title**: CUS-based deep learning for marker-free CTC identification

Bin Ye^1,2,3^, Zhen Wang^1^, Yinqi Bai^4^, Xu Zhang^5^, Rui Zhang^6^, Jingru Lian^3^, Xuefei Liu^3^, Yan Zhang^7^, Zhiyuan Xu^7^, Li Yang^7^, Haiman Jin^7^, Fang Chen^7^, Zhihao Xie^8^, Ping Zhou^8^, *Jun Tan*^9^, Shan Zeng^10^, Changzheng Du^3^, Yang Min^1^, Xiaomin Ni^1^, Jingxian Duan^1^, Zhicheng Li^1^, Hui Yang^1^, Yunpeng Cai^6^, Hongyan Wu^6^, Catherine C. Liu^11^, Jing Cai^12^, Yi Lu^13,14^, Jian Zhang^13,14,15^, Hao Wu^16^, Hairong Zheng^* 1,17^, Longqi Liu^* 4^, Xin Hong^* 3,18,19^, and Hao Yu^* 1,17,20^

^1^ Institute of Biomedical and Health Engineering, Shenzhen Institute of Advanced Technology, Chinese Academy of Sciences, Shenzhen 518055, China

^2^ School of Life Sciences and Medical Engineering, Anhui University, Hefei 230601, China

^3^ Department of Biochemistry, SUSTech Homeostatic Medicine Institute, School of Medicine, Southern University of Science and Technology, Shenzhen 518055, China

^4^ BGI-Research, Hangzhou 310000, China

^5^ School of Mathematical Sciences, South China Normal University, Guangzhou 510631, China

^6^ Research Center for Biomedical Information Technology, Shenzhen Institute of Advanced Technology, Chinese Academy of Sciences, Shenzhen 518055, China

^7^ Department of Clinical Oncology, University of Hong Kong-Shenzhen Hospital, Shenzhen 518053, China

^8^ Department of Radiotherapy, The First Affiliated Hospital of Hainan Medical University, Haikou 570208, China

^9^ Department of Neurosurgery, Xiangya Hospital, Central South University, Changsha 410028, China

^10^ Department of Oncology, Xiangya Hospital, Central South University, Changsha 410028, China

^11^ Department of Applied Mathematics, The Hong Kong Polytechnic University, Hong Kong 999077, China

^12^ Department of Health Technology and Informatics, The Hong Kong Polytechnic University, Hong Kong 999077, China

^13^ Joint Laboratory of Guangdong-Hong Kong Universities for Vascular Homeostasis and Diseases, SUSTech Homeostatic Medicine Institute, School of Medicine, Southern University of Science and Technology, Shenzhen 518055, China

^14^ Department of Human Cell Biology and Genetics, School of Medicine, Southern University of Science and Technology, Shenzhen 518055, China

^15^ Clinical Research Center, the First People's Hospital of Foshan (The Affiliated Foshan Hospital of Southern University of Science and Technology), School of Medicine, Southern University of Science and Technology, Shenzhen 528000, China

^16^ Faculty of Computer Science and Control Engineering, Shenzhen Institute of Advanced Technology, Chinese Academy of Sciences, Shenzhen 518055, China

^17^ The Key Laboratory of Biomedical Imaging Science and System, Chinese Academy of Sciences, Shenzhen 518055, China

^18^ Key University Laboratory of Metabolism and Health of Guangdong, Southern University of Science and Technology, Shenzhen 518055, China

^19^ Guangdong Provincial Key Laboratory of Cell Microenvironment and Disease Research, Southern University of Science and Technology, Shenzhen 518055, China.

^20^ Department of Biomedical Engineering, Shenzhen University of Advanced Technology, Shenzhen 518055, China

*Correspondence: [hao.yu@siat.ac.cn](mailto:hao.yu@siat.ac.cn) (Hao Yu); [liulongqi@genomics.cn](mailto:liulongqi@genomics.cn) (Longqi Liu); [hr.zheng@siat.ac.cn](mailto:hr.zheng@siat.ac.cn) (Hairong Zheng); [hongx@sustech.edu.cn](mailto:hongx@sustech.edu.cn) (Xin Hong)

**Supplementary Methods**

**Datasets used in this study**. We used public scRNA data from 12 solid primary cancer types, including CC, NSCLC, CRC, PDAC, NPC, EC, OVC, BC, PC, GC, HCC, and melanoma (Table S1). We newly sequenced primary CC tumor cells via 10X Genomics, and primary BC and CRC cells via Smart-seq in this study (Table S1). We also used public data on immune cells, including T cells, natural killer (NK) cells, monocytes, B cells, and dendritic cells, from healthy PBMCs (Table S2). We used public single CTCs or CTC clusters of 6 cancer types, including BC, NSCLC, PC, HCC, and melanoma, and newly sequenced CTCs from BC and CRC samples that were selected and validated by *ex vivo* experiments (Table S3). We also used public data on metastatic cancer cells, including those from melanoma, GC, and OVC (Table S4). We used scRNA-seq data from tumor-matched PBMCs of cancer patients, including public PBMCs (PDAC, CRC, NSCLC, GC, and NPC) and newly sequenced CC PBMCs, as well as public data from tumor-unmatched PBMCs of cancer patients (melanoma, OVC, HCC, and BC) (Table S5). Moreover, we used a PBMC dataset sequenced on the DNBelab-C4 platform (https://www.mgi-tech.com/) to analyze platform batch effects. Detailed information can be found in the Datasets used in the study and Data availability sections.

**Preparation of fresh tumor tissue and peripheral blood for 10X Genomics**. We constructed a cohort containing 6 cervical cancer patients with matched tumor tissues and PBMCs for scRNA sequencing via 10X Genomics. We collected matched PBMCs from the 6 patients by liquid biopsy and primary tumor samples by needle biopsy. These samples were collected from newly diagnosed cervical cancer patients with informed written consent and under approval by the Ethics Committee of the University of Hong Kong-Shenzhen Hospital (IRB NO. [2019]049-001) and The First Affiliated Hospital of Hainan Medical University (IRB NO. [2021]126). We immediately processed fresh tumor tissues and peripheral blood after biopsy to prepare single-cell suspensions. Tissue samples were cut into small pieces less than 1 mm in diameter and then digested using 2 mL of trypsin (Gibco, catalog number: R001100), 1 mL of collagenase II (Biofrox, Cat: 2275MG100), and 100 μL of DNase (Servicebio, Cat: 1121MG010) at 37°C for 1 hour on a shaker. Subsequently, the samples were suspended in 4 mL of 1640 culture medium and filtered through a 40 μm cell mesh. After washing twice with phosphate-buffered saline (PBS), the cells were resuspended in 1 mL of ice-cold erythrocyte lysis buffer and incubated for 10 min at 4°C. After washing twice with ice-cold PBS, the cells were counted with a hemocytometer under an inverted microscope. Trypan blue was used to confirm that the cell viability was at least 90%. Peripheral blood samples (5 mL) were collected with anticoagulant agents (heparin, EDTA, citrate, ACD-A or citrate phosphate dextrose) and diluted with 2–4 volumes of PBS (pH 7.2). We placed 10 mL of Ficoll-Paque PLUS density gradient medium (*ρ* = 1.077 g/mL) at the bottom of the tube with a 15 G hypodermic needle and then centrifuged the samples at 400$\times$g for 25 min at 20°C in a swinging bucket rotor without braking. Then, we removed the upper layer, carefully transferred the mononuclear cells at interphase to a new tube, added PBS, and mixed and centrifuged the tubes again at 300$\times$g for 10 min at 20°C. We removed the supernatant again, resuspended the cells in new PBS and centrifuged the mixture at 200$\times$g for 15 min at 20°C; this step was repeated twice to remove platelets. Finally, we counted the cells and preserved them in liquid nitrogen before sequencing.

**10X Genomics scRNA sequencing**. The 10X Genomics platform was used to perform scRNA sequencing of new samples from cervical cancer patients. New samples of primary tumor tissues and PBMCs were sequenced by Gene Denovo Ltd. via 10X Genomics Technology. Libraries were generated and sequenced from the cDNAs with Chromium Next GEM Single Cell 3’ Reagent Kits v3.1. Cell Ranger software 3.1.0 (https://www.10xgenomics.com/) was used to convert the raw BCL files to FASTQ files. Reads with low-quality barcodes and UMIs were removed, and the remaining reads were subsequently mapped to the hg19 reference genome generated from the hg19 genome sequence and the annotation file hg19.refGene.gtf in UCSC (https://hgdownload.cse.ucsc.edu/goldenPath/hg19/bigZips/). UMI counts and cell barcode calling were performed using the default parameters in Cell Ranger.

**Preprocessing of scRNA-seq data from 10X Genomics.** We performed quality control and doublet detection to filter out low-quality cells using the R package Seurat v4.0.5 [1]. Cells were filtered with a minimum of 200 genes and maximum numbers according to the distribution of gene numbers for each library; cells with a percentage of mitochondrial UMI count greater than 10%–30% were also discarded; and genes detected in fewer than 3 cells were removed. Then, we used the R package DoubletFinder v2.0.3 [2] to remove potential doublets with a doublet formation rate of 0.05 and automatically optimized neighborhood sizes for each library (Table S1). We performed feature selection using the FindVariableFeatures function of the Seurat package with the number of top variable features set to 2,000. We used the ScaleData function to scale the gene expression data and then performed principal component analysis (PCA). We used the R package Harmony v0.1.0 [3] to perform batch correction of samples from different libraries. We used the FindNeighbors and FindClusters functions with multiple resolutions to identify cell clusters and applied UMAP clustering algorithms to all the samples.

**Isolation of patient primary cancer cells and CTCs for Smart-seq.** The fresh tissue samples of a BC patient and a CRC patient were subsequently transported to the laboratory on ice. These samples were collected from newly diagnosed patients who provided informed written consent and according to the approval granted by the Ethics Committee of Xiangya Hospital (IRB NO.20210063). Upon arrival, the tissues were rinsed twice with PBS. Then, 50 µL of trypsin was added to each tube. The tissues were subsequently fragmented and lysed. The lysed tissues were then incubated at 37°C for 30 min with gentle agitation every 5 min. After the incubation, the suspension was filtered through a 70 µm filter. The filtrate was washed twice with PBS and stored for subsequent procedures. Patient CTCs were isolated by a spiral microfluidic device, and cell identity was confirmed by cell morphology. Then, the CTCs were cultured under *ex vivo* conditions as described previously [4, 5]. Cultured CTCs were ultimately selected by eliminating contaminating immune cells that were alive after less than 3 days.

**Sequencing and data preprocessing for Smart-seq.** Sorted CTCs and primary cancer cells from BC and CRC patients were processed using the Smart-seq3 protocol with minor changes [6]. scRNA-seq was generated in house. Sequencing was carried out with an average depth of 0.5 million reads per cell and 43 bp reads (single-end) after demultiplexing. After obtaining the sequencing FASTQ files, we generated BAM reads from FASTQ files using TopHat v2.1.0 [7] based on the UCSC hg19 reference genome with bowtie indices generated by Bowtie v2.1.0 [8]. Then, we generated the feature count matrix using the featureCounts program in subread v2.0.3 [9, 10] with default parameters.

**Cell annotation based on scRNA-seq data**. For the public data deposited with cell annotations, we used known cell information to directly select the target PBMC immune cells and cancer cells in different datasets. For the raw deposited data, we first performed data quality control, doublet removal, and basic cell annotation for public scRNA-seq data using methods identical to those used for fresh samples (see previous methods). To select cancer cells of different cancer types in different datasets, we traced the available biomarkers of cancer cells for every cancer type according to the original studies; for instance, *EPCAM*, *KRT18*, and *KRT19* were used for malignant epithelial cells in most cancer types; *ALB*, *SERPINA1*, and *HNF4A* were used for malignant cells in HCC [11]; *MUC16* and *WFDC2* were used for malignant cells in EC [12]; and *MLANA*, *PMEL*, and *MITF* were used for melanoma cells [13]. The T, NK, and B immune cells; monocytes; and DCs from PBMCs were selected according to their common specific biomarkers (T: *CD3D* and *CD3E*; NK: *NKG7* and *GNLY*; B: *CD79A* and *CD19*; monocytes: *FCGR3A* and *CD14*; DCs: *IRF8* and *MAP1A*). We then extracted the UMI count matrices of the immune or cancer cells in each library for downstream analysis.

**Analyses of scATAC-seq data**. We used scATAC-seq data from 10k_Human_PBMC_Multiome_v1.0 and Human_Kidney_Cancer_Nuclei accessed from https://www.10xgenomics.com/to extract chromatin accessible peaks at chromosome positions related to certain CUSs for T cells, B cells, NK cells, monocytes, DCs, and cancer cells. We used the Signac package [14] to analyze the single-cell chromatin state for each cell type. Then, we combined the peaks that were located within the range of chromosome positions for each CUS and calculated the mean as the ATAC mean peak for that CUS. We calculated Pearson’s correlations between the CUS value and the ATAC mean peak for each CUS.

**Comparison analysis**. To compare the CTC identification performance of panCTC with other methods, we generated pseudo-PBMCs by computationally combining scRNA-seq data from immune cells (from healthy donors) and cancer cells (including CTCs, primary/metastatic tumors) into validation datasets (Figure S6A). This approach provides ground-truth labels for benchmarking by simulating clinical PBMC samples, circumventing the current impossibility of *in vivo* CTC labeling. Immune cells were derived from healthy donor PBMCs and simulated to match physiological ratios (T: 73%, NK: 10%, monocyte: 10%, B: 5%, and DC: 2%) [15]. CTCs included single cells and clusters from: (1) *ex vivo* PBMCs of cancer patients, and (2) primary/metastatic tumor-derived cancer cells. To compare the CTC tracing accuracy of panCTC with other methods, we constructed 260 independent test datasets comprising varying quantities of CTCs and cancer cells derived from multiple sources (Figure S7A), including primary and metastatic tumors cells; single CTCs and CTC clusters isolated through *ex vivo* experiments; and CTCs identified by panCTC Model 1. All primary tumor classifications in these samples were previously confirmed by published studies and were rigorously excluded from model training. For methods CopyKat, scATOMIC, iCTC, and CTCTracer, their respective default manuals are used to make predictions based on the cell expression matrix. For two basic models, scFoundation and scGPT, high-dimensional embedding vectors are generated based on the expression matrix and used as input to construct a lightweight multi-layer perceptron (MLP) for task prediction. The training adopts the cross entropy loss function and enables early stopping based on the validation set loss (terminated if there is no improvement for 5 consecutive rounds), and finally evaluates the model performance on an independent test set.

**Differential expression gene analysis.** We identified gene expression markers of predicted CTCs versus all the other cells in the PBMC samples using the FindMarkers function of the Seurat package. The *p* values were adjusted according to the Bonferroni correction. We calculated the difference in the percentage of cells where the gene was detected in the CTCs versus all the other cells. We retained differentially expressed genes with a log-fold change > 0.6 and an adjusted *p* value < 0.05.

**Gene set enrichment analysis.** We performed enrichment function annotation analysis for genes associated with CUSs using DAVID (https://david.ncifcrf.gov/), selecting only significant KEGG pathways. We performed gene set enrichment analysis for predicted CTCs in the PBMC samples using clusterProfiler v4.2.0 [16] and GSEA v4.3.2 [17, 18]. By using the annotation data package org.Hs.eg.db v3.14.0 [19], we translated the symbols of the differentially expressed genes with a log-fold change > 0.6 of the predicted CTCs to the Ensembl ID by applying the bitr function. We then performed overrepresentation analysis using Gene Ontology (GO) by applying the enrichGO function. The redundant GO terms were removed by applying the simplify function with the cutoff set to 0.7 and selected features set to the minimum adjusted *p* values.

**Cell‒cell interaction analysis.** To determine the interactions between CTCs and circulating immune cells, we performed cell communication analysis using the R package CellChat v.1.1.3 [20]. We annotated the main immune cell types in each PBMC dataset. Since there were few CTCs in each dataset, we sampled at most 500 cells for each cell type and then detected the ligand–receptor pairs by using CTCs or immune cells as secreting cells.

**Estimation of the EMT score.** We computed the EMT score using the KSScore function [21] modified on the basis of a two-sample Kolmogorov–Smirnov test [22]. To apply this method, which was developed based on microarray bulk RNA data, to scRNA-seq data, we performed mapping between probe IDs and gene symbols using the R packages hgu133a.db [23] and annotate [24]. A positive score was associated with mesenchymal cells (M), whereas a negative score was associated with epithelial cells (E). We assessed normal epithelial cells [25], normal fibroblasts [26], and normal blood T cells (pbmc_10k_v3; https://www.10xgenomics.com) to compare the EMT scores of the CTCs.

**Survival analysis.** We used the TCGA and GEO datasets of ovarian cancer (https://kmplot.com/analysis/) to construct a Kaplan‒Meier survival plot for marker genes of CTCs identified from a new independent dataset of ovarian cancer [27]. The best cutoff value was determined with the lowest false discovery rate and the highest hazard rate using the Benjamini–Hochberg method [28].

**CNV inference and similarity estimation.** To visualize the malignancy status of the CTCs identified by panCTC, we performed CNV inference using the R package inferCNV v1.10.1 [29]. We defined healthy blood T cells and normal epithelial cells [25] as the reference cells. To determine the relationship between CTCs and primary cancer cells, we annotated the matched primary cancer cells to several subclusters at a resolution = 0.5 using the FindClusters function of the Seurat package (Figure S9). Because few CTCs were identified, we randomly sampled a few primary cancer cells to infer their CNV profiles. To compare the CNV profiles among CTCs and primary tumors, we used the R package CNVMetrics (https://github.com/krasnitzlab/CNVMetrics) to calculate similarities among CTCs and subclusters of primary tumors.

**Variant calling and somatic mutation analysis.** We performed mutation analysis for different cell groups based on scRNA-seq data using the workflow gatk4-rnaseq-germline-snps-indels (https://github.com/gatk-workflows/gatk4-rnaseq-germline-snps-indels) with GATK v4.3.0.0 [30] and STAR v2.7.4a [31]. We used the genome sequence of hg19 and annotation files of hg19.refGene.gtf in UCSC (https://hgdownload.cse.ucsc.edu/goldenPath/hg19/bigZips/), the same as that used in scRNA data preprocessing for fresh samples, to generate a reference genome using the STAR command --runMode genomeGenerate --sjdbOverhang 100. We downloaded resource files containing dbsnp138, known indels, and standard.indels.b37.sites from the Homo_sapiens_assembly19_1000genomes at gs://gcp-public-data--broad-references.

For the Smart-seq scRNA data, we first generated BAM reads from FASTQ files using TopHat v2.1.0 [7] based on the UCSC hg19 reference genome with bowtie indices generated with Bowtie v2.1.0 [8]. We subsequently mapped the BAM reads to the reference genome to obtain the output BAM files with duplicate reads marked and recalibrated by applying SAMtoFastq, which was subsequently aligned with STAR with “--sjdbOverhang 100 --outSAMtype BAM SortedByCoordinate”, AddOrReplaceReadGroups, MarkDuplicates, SplitNCigarReads, BaseRecalibrator, and ApplyBQSR implemented in GATK [32]. Then, we directly used the BAM files with duplicate reads marked to call the variants of CTCs and primary cancer cells from Smart-seq.

Subsequently, we sequentially ran HaplotypeCaller with the command --standard-min-confidence-threshold-for-calling 20, MergeVCFs with default, and VariantFiltration with command --segment 35 --cluster 3 --filter-name “FS” --filter “FS > 30.0” --filter-name “QD” --filter “QD < 2.0” implemented in GATK to perform VCF calling for each BAM file. Then, we ran the Funcotator implemented in GATK based on germline mutation data sources to annotate the mutations. To identify the somatic mutations in CTCs and primary cancer cells and reveal the correlations between them, we applied 539 somatic mutation genes (v98, May 2023) from Tier 1 of the Cancer Gene Census [33] in the COSMIC database (https://cancer.sanger.ac.uk/census) to filter the somatic mutation variants for CTCs and primary cancer cells. Finally, we used the R package maftools v2.10.05 [34] to summarize the results.

**Datasets used in this study.**

The public scRNA-seq data of primary cancer cells were obtained from the GEO database (accession nos. GSE155698 [35], GSE149614 [11], GSE173682 [12], GSE215120 [13], GSE168652 [25], GSE193337 [36], GSE176031 [37], GSE178318 [38], GSE132465 [39], GSE162025 [40], GSE150825 [41], GSE150430 [42], GSE176078 [43], GSE127465 [44], GSE148071 [45], GSM3946506 [46]), the ArrayExpress database (accession no. E-MTAB-11948 [47]), and the Open Archive for Miscellaneous Data database (accession no. OMIX001073 [48]). The publicly available scRNA-seq data of healthy PBMCs were obtained from the GSE155698 [35], GSE199994 [49], 10X Genomics pbmc_10k_v3 (https://www.10xgenomics.com), and 10X Genomics pbmc_68k [50] datasets and from Integrated Analysis of Mutilmodal Single-Cell Data (https://atlas.fredhutch.org/nygc/multimodal-pbmc/), GSE149689 [51], GSE150728 [52], GSE168732 [53], and the CNGB Nucleotide Sequence Archive NO. CNP0001102 [54]. The publicly available scRNA-seq data for CTCs were obtained from the GSE75367 [55], GSE144494 [56], GSE144495 [56], GSE86978 [57], and GSE51827 [58] cohorts and from the GSE55807 [59], GSE157740 [5], GSE157743 [5], GSE74639 [60], GSE67980 [61], and CNGB Nucleotide Sequence Archive NO. CNP0000095[62]. The public scRNA-seq data of metastatic cancer cells were obtained from the GSE176029 [63], GSE163558 [64], and GSE147082 datasets [65]. Public scRNA data of patient PBMCs matched with tumor are GSE155698 [35], GSE178318 [38], GSE162025 [40], GSE127465 [44], and OMIX001073 [48]; Public scRNA data of patient PBMCs unmatched with tumor are GSE189125[66], GSE213243 [67], GSE107747 [68], and GSE174463 [69]. The public scRNA-seq data of normal fibroblasts and epithelial cells used in the analyses were obtained from the GSE163973 [26] and GSE168652 cohorts, respectively [25]. Public scRNA-seq data from ovarian cancer patients were obtained from https://doi.org/10.17632/rc47y6m9mp.1 (PRJCA005422) [27] for clinical analysis of CTCs. Public scATAC-seq data for 10k_Human_PBMC_Multiome_v1.0 and Human_Kidney_Cancer_Nuclei were obtained from https://www.10xgenomics.com/.

**REFERENCES**

1. Hao, Yuhan, Stephanie Hao, Erica Andersen-Nissen, William M. Mauck, III, Shiwei Zheng, Andrew Butler, Maddie J. Lee, et al. 2021. “Integrated analysis of multimodal single-cell data.” *Cell* 184: 3573–3587. <https://doi.org/10.1016/j.cell.2021.04.048>

2. McGinnis, Christopher S., Lyndsay M. Murrow, Zev J. Gartner. 2019. “DoubletFinder: Doublet detection in single-cell RNA sequencing data using artificial nearest neighbors.” *Cell Systems* 8: 329–337. <https://doi.org/10.1016/j.cels.2019.03.003>

3. Korsunsky, Ilya, Nghia Millard, Jean Fan, Kamil Slowikowski, Fan Zhang, Kevin Wei, Yuriy Baglaenko, Michael Brenner, Po-ru Loh, Soumya Raychaudhuri. 2019. “Fast, sensitive and accurate integration of single-cell data with Harmony.” *Nature methods* 16: 1289–1296. <https://doi.org/10.1038/s41592-019-0619-0>

4. Warkiani, Majid Ebrahimi, Bee Luan Khoo, Lidan Wu, Andy Kah Ping Tay, Ali Asgar S. Bhagat, Jongyoon Han, Chwee Teck Lim. 2016. “Ultra-fast, label-free isolation of circulating tumor cells from blood using spiral microfluidics.” *Nature Protocols* 11: 134–148. <https://doi.org/10.1038/nprot.2016.003>

5. Hong, Xin, Whijae Roh, Ryan J. Sullivan, Keith H. K. Wong, Ben S. Wittner, Hongshan Guo, Taronish D. Dubash, et al. 2021. “The lipogenic regulator SREBP2 induces transferrin in circulating melanoma cells and suppresses ferroptosis.” *Cancer Discovery* 11: 678–695. <https://doi.org/10.1158/2159-8290.CD-19-1500>

6. Hagemann-Jensen, Michael, Christoph Ziegenhain, Ping Chen, Daniel Ramsköld, Gert-Jan Hendriks, Anton J. M. Larsson, Omid R. Faridani, Rickard Sandberg. 2020. “Single-cell RNA counting at allele and isoform resolution using Smart-seq3.” *Nature Biotechnology* 38: 708–714. <https://doi.org/10.1038/s41587-020-0497-0>

7. Kim, Daehwan, Geo Pertea, Cole Trapnell, Harold Pimentel, Ryan Kelley, Steven L. Salzberg. 2013. “TopHat2: accurate alignment of transcriptomes in the presence of insertions, deletions and gene fusions.” *Genome Biology* 14: R36. <https://doi.org/10.1186/gb-2013-14-4-r36>

8. Langmead, Ben, Cole Trapnell, Mihai Pop, Steven L. Salzberg. 2009. “Ultrafast and memory-efficient alignment of short DNA sequences to the human genome.” *Genome Biology* 10: R25. <https://doi.org/10.1186/gb-2009-10-3-r25>

9. Liao, Yang, Gordon K. Smyth, Wei Shi. 2013. “The Subread aligner: fast, accurate and scalable read mapping by seed-and-vote.” *Nucleic Acids Research* 41: e108–e108. <https://doi.org/10.1093/nar/gkt214>

10. Liao, Yang, Gordon K. Smyth, Wei Shi. 2014. “featureCounts: an efficient general purpose program for assigning sequence reads to genomic features.” *Bioinformatics* 30: 923–930. <https://doi.org/10.1093/bioinformatics/btt656>

11. Lu, Yiming, Aiqing Yang, Cheng Quan, Yingwei Pan, Haoyun Zhang, Yuanfeng Li, Chengming Gao, et al. 2022. “A single-cell atlas of the multicellular ecosystem of primary and metastatic hepatocellular carcinoma.” *Nature Communications* 13: 4594. <https://doi.org/10.1038/s41467-022-32283-3>

12. Regner, Matthew J., Kamila Wisniewska, Susana Garcia-Recio, Aatish Thennavan, Raul Mendez-Giraldez, Venkat S. Malladi, Gabrielle Hawkins, et al. 2021. “A multi-omic single-cell landscape of human gynecologic malignancies.” *Molecular Cell* 81: 4924–4941. <https://doi.org/10.1016/j.molcel.2021.10.013>

13. Zhang, Chao, Hongru Shen, Tielong Yang, Ting Li, Xinyue Liu, Jin Wang, Zhichao Liao, et al. 2022. “A single-cell analysis reveals tumor heterogeneity and immune environment of acral melanoma.” *Nature Communications* 13: 7250. <https://doi.org/10.1038/s41467-022-34877-3>

14. Stuart, Tim, Avi Srivastava, Shaista Madad, Caleb A. Lareau, Rahul Satija. 2021. “Single-cell chromatin state analysis with Signac.” *Nature methods* 18: 1333–1341. <https://doi.org/10.1038/s41592-021-01282-5>

15. Kleiveland, Charlotte R. 2015. Peripheral blood mononuclear cells. *The Impact of Food Bioactives on Health: in vitro and ex vivo models* Springer International Publishing, 161–167. <https://doi.org/10.1007/978-3-319-16104-4_15>

16. Wu, Tianzhi, Erqiang Hu, Shuangbin Xu, Meijun Chen, Pingfan Guo, Zehan Dai, Tingze Feng, et al. 2021. “clusterProfiler 4.0: A universal enrichment tool for interpreting omics data.” *The Innovation* 2: 100141. <https://doi.org/10.1016/j.xinn.2021.100141>

17. Mootha, Vamsi K., Cecilia M. Lindgren, Karl-Fredrik Eriksson, Aravind Subramanian, Smita Sihag, Joseph Lehar, Pere Puigserver, et al. 2003. “PGC-1α-responsive genes involved in oxidative phosphorylation are coordinately downregulated in human diabetes.” *Nature Genetics* 34: 267–273. <https://doi.org/10.1038/ng1180>

18. Subramanian, Aravind, Pablo Tamayo, Vamsi K. Mootha, Sayan Mukherjee, Benjamin L. Ebert, Michael A. Gillette, Amanda Paulovich, et al. 2005. “Gene set enrichment analysis: A knowledge-based approach for interpreting genome-wide expression profiles.” *Proceedings of the National Academy of Sciences* 102: 15545–15550. <https://doi.org/10.1073/pnas.0506580102>

19. Carlson, M. 2021. “org.Hs.eg.db: Genome wide annotation for Human.” *R package version 3.14.0*,

20. Jin, Suoqin, Christian F. Guerrero-Juarez, Lihua Zhang, Ivan Chang, Raul Ramos, Chen-Hsiang Kuan, Peggy Myung, Maksim V. Plikus, Qing Nie. 2021. “Inference and analysis of cell-cell communication using CellChat.” *Nature Communications* 12: 1088. <https://doi.org/10.1038/s41467-021-21246-9>

21. Chakraborty, Priyanka, Jason T. George, Shubham Tripathi, Herbert Levine, Mohit Kumar Jolly. 2020. “Comparative study of transcriptomics-based scoring metrics for the epithelial-hybrid-mesenchymal spectrum.” *Frontiers in Bioengineering and Biotechnology* 8: 220. <https://doi.org/10.3389/fbioe.2020.00220>

22. Tan, Tuan Zea, Qing Hao Miow, Yoshio Miki, Tetsuo Noda, Seiichi Mori, Ruby Yun-Ju Huang, Jean Paul Thiery. 2014. “Epithelial-mesenchymal transition spectrum quantification and its efficacy in deciphering survival and drug responses of cancer patients.” *EMBO Molecular Medicine* 6: 1279–1293. <https://doi.org/10.15252/emmm.201404208>

23. Carlson, M. 2021. “hgu133a.db: Affymetrix HG-U133A Array annotation data (chip hgu133a).” *R package version 3.13.0*,

24. Gentleman, R. 2021. “annotate: Annotation for microarrays.” *R package version 1.72.0*,

25. Li, Chunbo, Luopei Guo, Shengli Li, Keqin Hua. 2021. “Single-cell transcriptomics reveals the landscape of intra-tumoral heterogeneity and transcriptional activities of ECs in CC.” *Molecular Therapy - Nucleic Acids* 24: 682–694. <https://doi.org/10.1016/j.omtn.2021.03.017>

26. Deng, Cheng-Cheng, Yong-Fei Hu, Ding-Heng Zhu, Qing Cheng, Jing-Jing Gu, Qing-Lan Feng, Li-Xue Zhang, et al. 2021. “Single-cell RNA-seq reveals fibroblast heterogeneity and increased mesenchymal fibroblasts in human fibrotic skin diseases.” *Nature Communications* 12: 3709. <https://doi.org/10.1038/s41467-021-24110-y>

27. Zheng, Xiaocui, Xinjing Wang, Xi Cheng, Zhaoyuan Liu, Yujia Yin, Xiaoduan Li, Zhihao Huang, et al. 2023. “Single-cell analyses implicate ascites in remodeling the ecosystems of primary and metastatic tumors in ovarian cancer.” *Nature Cancer* 4: 1138–1156. <https://doi.org/10.1038/s43018-023-00599-8>

28. Győrffy, Balázs. 2023. “Discovery and ranking of the most robust prognostic biomarkers in serous ovarian cancer.” *GeroScience* 45: 1889–1898. <https://doi.org/10.1007/s11357-023-00742-4>

29. Tickle, T.I., C. Georgescu, M. Brown, B. Haas. 2019. “inferCNV of the Trinity CTAT Project. <https://github.com/broadinstitute/inferCNV>.” <https://github.com/broadinstitute/inferCNV>

30. McKenna, Aaron, Matthew Hanna, Eric Banks, Andrey Sivachenko, Kristian Cibulskis, Andrew Kernytsky, Kiran Garimella, et al. 2010. “The Genome Analysis Toolkit: A MapReduce framework for analyzing next-generation DNA sequencing data.” *Genome Research* 20: 1297–1303. <https://doi.org/10.1101/gr.107524.110>

31. Dobin, Alexander, Carrie A. Davis, Felix Schlesinger, Jorg Drenkow, Chris Zaleski, Sonali Jha, Philippe Batut, Mark Chaisson, Thomas R. Gingeras. 2013. “STAR: ultrafast universal RNA-seq aligner.” *Bioinformatics* 29: 15–21. <https://doi.org/10.1093/bioinformatics/bts635>

32. Sharma, Ankur, Elaine Yiqun Cao, Vibhor Kumar, Xiaoqian Zhang, Hui Sun Leong, Angeline Mei Lin Wong, Neeraja Ramakrishnan, et al. 2018. “Longitudinal single-cell RNA sequencing of patient-derived primary cells reveals drug-induced infidelity in stem cell hierarchy.” *Nature Communications* 9: 4931. <https://doi.org/10.1038/s41467-018-07261-3>

33. Sondka, Zbyslaw, Sally Bamford, Charlotte G. Cole, Sari A. Ward, Ian Dunham, Simon A. Forbes. 2018. “The COSMIC Cancer Gene Census: describing genetic dysfunction across all human cancers.” *Nature Reviews Cancer* 18: 696–705. <https://doi.org/10.1038/s41568-018-0060-1>

34. Mayakonda, Anand, De-Chen Lin, Yassen Assenov, Christoph Plass, H. Phillip Koeffler. 2018. “Maftools: efficient and comprehensive analysis of somatic variants in cancer.” *Genome Research* 28: 1747–1756. <https://doi.org/10.1101/gr.239244.118>

35. Steele, Nina G., Eileen S. Carpenter, Samantha B. Kemp, Veerin R. Sirihorachai, Stephanie The, Lawrence Delrosario, Jenny Lazarus, et al. 2020. “Multimodal mapping of the tumor and peripheral blood immune landscape in human pancreatic cancer.” *Nature Cancer* 1: 1097–1112. <https://doi.org/10.1038/s43018-020-00121-4>

36. Heidegger, Isabel, Georgios Fotakis, Anne Offermann, Jermaine Goveia, Sophia Daum, Stefan Salcher, Asma Noureen, et al. 2022. “Comprehensive characterization of the prostate tumor microenvironment identifies CXCR4/CXCL12 crosstalk as a novel antiangiogenic therapeutic target in prostate cancer.” *Molecular Cancer* 21: 132. <https://doi.org/10.1186/s12943-022-01597-7>

37. Song, Hanbing, Hannah N. W. Weinstein, Paul Allegakoen, Marc H. Wadsworth, Jamie Xie, Heiko Yang, Ethan A. Castro, et al. 2022. “Single-cell analysis of human primary prostate cancer reveals the heterogeneity of tumor-associated epithelial cell states.” *Nature Communications* 13: 141. <https://doi.org/10.1038/s41467-021-27322-4>

38. Che, Li-Heng, Jing-Wen Liu, Jian-Ping Huo, Rong Luo, Rui-Ming Xu, Cai He, Yu-Qing Li, et al. 2021. “A single-cell atlas of liver metastases of colorectal cancer reveals reprogramming of the tumor microenvironment in response to preoperative chemotherapy.” *Cell Discovery* 7: 80. <https://doi.org/10.1038/s41421-021-00312-y>

39. Lee, Hae-Ock, Yourae Hong, Hakki Emre Etlioglu, Yong Beom Cho, Valentina Pomella, Ben Van den Bosch, Jasper Vanhecke, et al. 2020. “Lineage-dependent gene expression programs influence the immune landscape of colorectal cancer.” *Nature Genetics* 52: 594–603. <https://doi.org/10.1038/s41588-020-0636-z>

40. Liu, Yang, Shuai He, Xi-Liang Wang, Wan Peng, Qiu-Yan Chen, Dong-Mei Chi, Jie-Rong Chen, et al. 2021. “Tumour heterogeneity and intercellular networks of nasopharyngeal carcinoma at single cell resolution.” *Nature Communications* 12: 741. <https://doi.org/10.1038/s41467-021-21043-4>

41. Gong, Lanqi, Dora Lai-Wan Kwong, Wei Dai, Pingan Wu, Shanshan Li, Qian Yan, Yu Zhang, et al. 2021. “Comprehensive single-cell sequencing reveals the stromal dynamics and tumor-specific characteristics in the microenvironment of nasopharyngeal carcinoma.” *Nature Communications* 12: 1540. <https://doi.org/10.1038/s41467-021-21795-z>

42. Chen, Yu-Pei, Jian-Hua Yin, Wen-Fei Li, Han-Jie Li, Dong-Ping Chen, Cui-Juan Zhang, Jia-Wei Lv, et al. 2020. “Single-cell transcriptomics reveals regulators underlying immune cell diversity and immune subtypes associated with prognosis in nasopharyngeal carcinoma.” *Cell Research* 30: 1024–1042. <https://doi.org/10.1038/s41422-020-0374-x>

43. Papanicolaou, Michael, Amelia L. Parker, Michelle Yam, Elysse C. Filipe, Sunny Z. Wu, Jessica L. Chitty, Kaitlin Wyllie, et al. 2022. “Temporal profiling of the breast tumour microenvironment reveals collagen XII as a driver of metastasis.” *Nature Communications* 13: 4587. <https://doi.org/10.1038/s41467-022-32255-7>

44. Zilionis, Rapolas, Camilla Engblom, Christina Pfirschke, Virginia Savova, David Zemmour, Hatice D. Saatcioglu, Indira Krishnan, et al. 2019. “Single-cell transcriptomics of human and mouse lung cancers reveals conserved myeloid populations across individuals and species.” *Immunity* 50: 1317–1334. <https://doi.org/10.1016/j.immuni.2019.03.009>

45. Wu, Fengying, Jue Fan, Yayi He, Anwen Xiong, Jia Yu, Yixin Li, Yan Zhang, et al. 2021. “Single-cell profiling of tumor heterogeneity and the microenvironment in advanced non-small cell lung cancer.” *Nature Communications* 12: 2540. <https://doi.org/10.1038/s41467-021-22801-0>

46. Wouters, Jasper, Zeynep Kalender-Atak, Liesbeth Minnoye, Katina I. Spanier, Maxime De Waegeneer, Carmen Bravo González-Blas, David Mauduit, et al. 2020. “Robust gene expression programs underlie recurrent cell states and phenotype switching in melanoma.” *Nature Cell Biology* 22: 986–998. <https://doi.org/10.1038/s41556-020-0547-3>

47. Li, Chunbo, Hao Wu, Luopei Guo, Danyang Liu, Shimin Yang, Shengli Li, Keqin Hua. 2022. “Single-cell transcriptomics reveals cellular heterogeneity and molecular stratification of cervical cancer.” *Communications Biology* 5: 1208. <https://doi.org/10.1038/s42003-022-04142-w>

48. Sun, Keyong, Runda Xu, Fuhai Ma, Naixue Yang, Yang Li, Xiaofeng Sun, Peng Jin, et al. 2022. “scRNA-seq of gastric tumor shows complex intercellular interaction with an alternative T cell exhaustion trajectory.” *Nature Communications* 13: 4943. <https://doi.org/10.1038/s41467-022-32627-z>

49. Boukhaled, Giselle M., Ramy Gadalla, Heidi J. Elsaesser, Diala Abd-Rabbo, Rene Quevedo, S. Y. Cindy Yang, Mengdi Guo, et al. 2022. “Pre-encoded responsiveness to type I interferon in the peripheral immune system defines outcome of PD1 blockade therapy.” *Nature Immunology* 23: 1273–1283. <https://doi.org/10.1038/s41590-022-01262-7>

50. Zheng, Grace X. Y., Jessica M. Terry, Phillip Belgrader, Paul Ryvkin, Zachary W. Bent, Ryan Wilson, Solongo B. Ziraldo, et al. 2017. “Massively parallel digital transcriptional profiling of single cells.” *Nature Communications* 8: 14049. <https://doi.org/10.1038/ncomms14049>

51. Lee, J. S., S. Park, H. W. Jeong, J. Y. Ahn, S. J. Choi, H. Lee, B. Choi, et al. 2020. “Immunophenotyping of COVID-19 and influenza highlights the role of type I interferons in development of severe COVID-19.” *Science Immunology* 5: eabd1554. <https://doi.org/10.1126/sciimmunol.abd1554>

52. Wilk, Aaron J., Arjun Rustagi, Nancy Q. Zhao, Jonasel Roque, Giovanny J. Martínez-Colón, Julia L. McKechnie, Geoffrey T. Ivison, et al. 2020. “A single-cell atlas of the peripheral immune response in patients with severe COVID-19.” *Nature Medicine* 26: 1070–1076. <https://doi.org/10.1038/s41591-020-0944-y>

53. Wang, Zhen, Lijian Xie, Guohui Ding, Sirui Song, Liqin Chen, Guang Li, Min Xia, et al. 2021. “Single-cell RNA sequencing of peripheral blood mononuclear cells from acute Kawasaki disease patients.” *Nature Communications* 12: 5444. <https://doi.org/10.1038/s41467-021-25771-5>

54. Zhu, Linnan, Penghui Yang, Yingze Zhao, Zhenkun Zhuang, Zhifeng Wang, Rui Song, Jie Zhang, et al. 2020. “Single-cell sequencing of peripheral mononuclear cells reveals distinct immune response landscapes of COVID-19 and influenza patients.” *Immunity* 53: 685–696. <https://doi.org/10.1016/j.immuni.2020.07.009>

55. Jordan, Nicole Vincent, Aditya Bardia, Ben S. Wittner, Cyril Benes, Matteo Ligorio, Yu Zheng, Min Yu, et al. 2016. “HER2 expression identifies dynamic functional states within circulating breast cancer cells.” *Nature* 537: 102–106. <https://doi.org/10.1038/nature19328>

56. Ebright, Richard Y., Sooncheol Lee, Ben S. Wittner, Kira L. Niederhoffer, Benjamin T. Nicholson, Aditya Bardia, Samuel Truesdell, et al. 2020. “Deregulation of ribosomal protein expression and translation promotes breast cancer metastasis.” *Science* 367: 1468–1473. <https://doi.org/10.1126/science.aay0939>

57. Aceto, Nicola, Aditya Bardia, Ben S. Wittner, Maria C. Donaldson, Ryan O'Keefe, Amanda Engstrom, Francesca Bersani, et al. 2018. “AR Expression in breast cancer CTCs associates with bone metastases.” *Molecular cancer research : MCR* 16: 720–727. <https://doi.org/10.1158/1541-7786.MCR-17-0480>

58. Aceto, Nicola, Aditya Bardia, David T Miyamoto, Maria C Donaldson, Ben S Wittner, Joel A Spencer, Min Yu, et al. 2014. “Circulating tumor cell clusters are oligoclonal precursors of breast cancer metastasis.” *Cell* 158: 1110–1122. <https://doi.org/10.1016/j.cell.2014.07.013>

59. Yu, Min, Aditya Bardia, Nicola Aceto, Francesca Bersani, Marissa W. Madden, Maria C. Donaldson, Rushil Desai, et al. 2014. “Ex vivo culture of circulating breast tumor cells for individualized testing of drug susceptibility.” *Science* 345: 216–220. <https://doi.org/10.1126/science.1253533>

60. Zheng, Yu, David T. Miyamoto, Ben S. Wittner, James P. Sullivan, Nicola Aceto, Nicole Vincent Jordan, Min Yu, et al. 2017. “Expression of β-globin by cancer cells promotes cell survival during blood-borne dissemination.” *Nature Communications* 8: 14344. <https://doi.org/10.1038/ncomms14344>

61. Miyamoto, David T., Yu Zheng, Ben S. Wittner, Richard J. Lee, Huili Zhu, Katherine T. Broderick, Rushil Desai, et al. 2015. “RNA-Seq of single prostate CTCs implicates noncanonical Wnt signaling in antiandrogen resistance.” *Science* 349: 1351–1356. <https://doi.org/10.1126/science.aab0917>

62. Sun, Yun-Fan, Liang Wu, Shi-Ping Liu, Miao-Miao Jiang, Bo Hu, Kai-Qian Zhou, Wei Guo, et al. 2021. “Dissecting spatial heterogeneity and the immune-evasion mechanism of CTCs by single-cell RNA-seq in hepatocellular carcinoma.” *Nature Communications* 12: 4091. <https://doi.org/10.1038/s41467-021-24386-0>

63. Lin, Weitao, Aaron B. Beasley, Nima Mesbah Ardakani, Elena Denisenko, Leslie Calapre, Matthew Jones, Benjamin A. Wood, Lydia Warburton, Alistair R. R. Forrest, Elin S. Gray. 2021. “Intra- and intertumoral heterogeneity of liver metastases in a patient with uveal melanoma revealed by single-cell RNA sequencing.” *Molecular Case Studies* 7: a006111. <https://doi.org/10.1101/mcs.a006111>

64. Jiang, Haiping, Dingyi Yu, Penghui Yang, Rongfang Guo, Mei Kong, Yuan Gao, Xiongfei Yu, Xiaoyan Lu, Xiaohui Fan. 2022. “Revealing the transcriptional heterogeneity of organ-specific metastasis in human gastric cancer using single-cell RNA Sequencing.” *Clinical and Translational Medicine* 12: e730. <https://doi.org/10.1002/ctm2.730>

65. Olalekan, Susan, Bingqing Xie, Rebecca Back, Heather Eckart, Anindita Basu. 2021. “Characterizing the tumor microenvironment of metastatic ovarian cancer by single-cell transcriptomics.” *Cell Reports* 35: 109165. <https://doi.org/10.1016/j.celrep.2021.109165>

66. Lozano, Alexander X., Aadel A. Chaudhuri, Aishwarya Nene, Antonietta Bacchiocchi, Noah Earland, Matthew D. Vesely, Abul Usmani, et al. 2022. “T cell characteristics associated with toxicity to immune checkpoint blockade in patients with melanoma.” *Nature Medicine* 28: 353–362. <https://doi.org/10.1038/s41591-021-01623-z>

67. Ren, Yanyu, Runrong Li, Hanxiao Feng, Jieying Xie, Lin Gao, Shuai Chu, Yan Li, Fanliang Meng, Yunshan Ning. 2022. “Single-cell sequencing reveals effects of chemotherapy on the immune landscape and TCR/BCR clonal expansion in a relapsed ovarian cancer patient.” *Frontiers in Immunology* 13: 985187. <https://doi.org/10.3389/fimmu.2022.985187>

68. D’Avola, Delia, Carlos Villacorta-Martin, Sebastiao N. Martins-Filho, Amanda Craig, Ismail Labgaa, Johann von Felden, Allette Kimaada, et al. 2018. “High-density single cell mRNA sequencing to characterize circulating tumor cells in hepatocellular carcinoma.” *Scientific Reports* 8: 11570. <https://doi.org/10.1038/s41598-018-30047-y>

69. Pauken, Christine M., Shelby R. Kenney, Kathryn J. Brayer, Yan Guo, Ursa A. Brown-Glaberman, Dario Marchetti. 2021. “Heterogeneity of circulating tumor cell neoplastic subpopulations outlined by single-cell transcriptomics.” *Cancers* 13: 4885. <https://doi.org/10.3390/cancers13194885>

**Supplementary Text S1:**

**Overview of the panCTC framework**

We developed panCTC, a comprehensive computational framework called for the identification of circulating tumor cells (CTCs) from single-cell RNA sequencing (scRNA-seq) data of peripheral blood mononuclear cells (PBMCs). The training of the panCTC framework comprised two key stages: First, a cell-by-CUS matrix was derived from unique molecular identifier (UMI) counts using scRNA-seq data derived from primary tumor tissues (Figure S1B). Second, hierarchical artificial intelligence (AI) models were trained on this CUS matrix—initially to distinguish CTCs from PBMCs, and subsequently to predict their tumor tissue of origin (Figure S1C).

1. **Converting the UMI count matrix into the CUS matrix**
   1. **Calculating the prior distribution of gene expression in each cell**

The workflow of deriving the CUS matrix from the UMI matrix is illustrated in Figure S1B. For UMI count matrix, each raw UMI count [
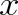
](https://www.codecogs.com/eqnedit.php?latex=x#0) is first square root transformed using Freeman-Tukey transformation and then log transformed, we denote the transformed count as [
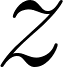
](https://www.codecogs.com/eqnedit.php?latex=z#0), then:

.

All genes are arranged according to their genomic coordinates (GENCODE Release 103), and connected to the sequence [
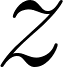
](https://www.codecogs.com/eqnedit.php?latex=z#0) with 33,575 gene expression values in length. We denote the first gene as [
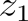
](https://www.codecogs.com/eqnedit.php?latex=z_1#0), and the last one as [
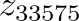
](https://www.codecogs.com/eqnedit.php?latex=z_%7B33575%7D#0). We implement the polynomial dynamic linear modeling (DLM) to smooth the gene expressions along the genome. When considering the polynomial model of order one as we only include the RNA expression data, the DLM is specified as:

[
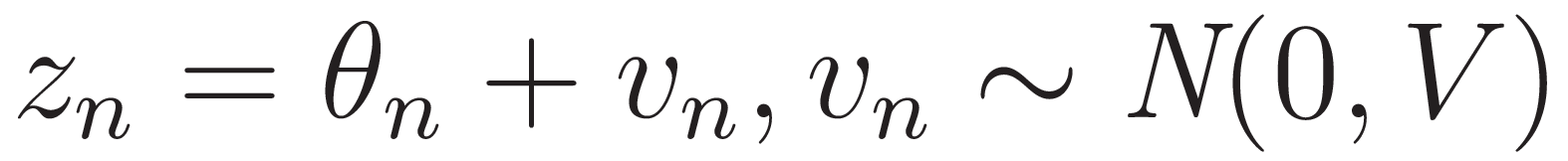
](http://www.sciweavers.org/tex2img.php?bc=Transparent&fc=Black&im=jpg&fs=100&ff=modern&edit=0&eq=z_n%3D%5Ctheta%20_n%2Bv_n%2C%20v_n%5Csim%20%5Ctextit%7BN%7D(0%2CV)%09#0),

[
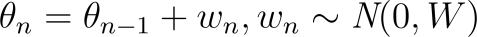
](https://www.codecogs.com/eqnedit.php?latex=%5Ctheta%20_n%3D%5Ctheta%20_%7Bn-1%7D%2Bw_n%2C%20w_n%5Csim%20%5Ctextit%7BN%7D(0%2CW)#0).

where [
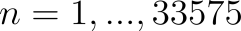
](https://www.codecogs.com/eqnedit.php?latex=n%3D1%2C...%2C33575#0), and [
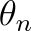
](https://www.codecogs.com/eqnedit.php?latex=%5Ctheta_n#0) is an unobservable state variable, with a prior distribution for the initial state [
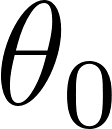
](https://www.codecogs.com/eqnedit.php?latex=%5Ctheta_0#0). [
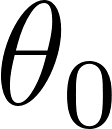
](https://www.codecogs.com/eqnedit.php?latex=%5Ctheta_0#0) is assumed to follow a normal distribution, with mean [
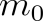
](https://www.codecogs.com/eqnedit.php?latex=m_0#0) (set to 0, be default) and variance [
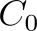
](https://www.codecogs.com/eqnedit.php?latex=C_0#0) (set to 1e+07, by default). The sequences [
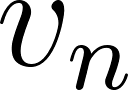
](https://www.codecogs.com/eqnedit.php?latex=v_n#0) and [
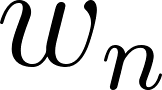
](https://www.codecogs.com/eqnedit.php?latex=w_n#0) are two noise terms, assumed to be independent of each other, both following the normal distribution with mean 0 and respective variances [
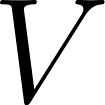
](https://www.codecogs.com/eqnedit.php?latex=V#0) and [
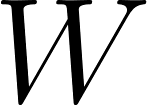
](https://www.codecogs.com/eqnedit.php?latex=W#0). Additionally, [
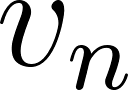
](https://www.codecogs.com/eqnedit.php?latex=v_n#0), [
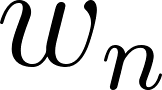
](https://www.codecogs.com/eqnedit.php?latex=w_n#0), and [
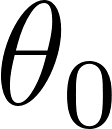
](https://www.codecogs.com/eqnedit.php?latex=%5Ctheta_0#0) are also independent of each other.

The equation set is solved by functions ‘dlmModPoly()’ and ‘dlmSmooth()’ from R package ***dlm***, with the variance [
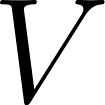
](https://www.codecogs.com/eqnedit.php?latex=V#0) set to 0.16 and [
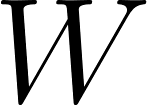
](https://www.codecogs.com/eqnedit.php?latex=W#0) set to 0.001. The modeled expression for each gene is used as the smoothed observed counts to update the parameters of the posterior distribution in the downstream Bayesian inference process.

- 1. **Calculating the posterior mean of gene expression in each CUS of a cell**

For each CUS, there are a number of genes (10 genes, by default) sequentially expressed. Therefore, we aim to integrate these expressions into a single value to represent the overall expression level for one CUS. Instead of directly calculating the mean of these genes, we model the gene expressions in one CUS using the negative-binomial distribution to accommodate the overdispersion issues in droplet scRNA-seq data. Since the negative-binomial distribution can be alternatively formulated as a Poisson-gamma mixture, we establish a Monte Carlo simulation based on a Poisson likelihood with a gamma prior to generate the posterior mean of the CUS. using the smoothed observed counts calculated in the last section (denoted as [
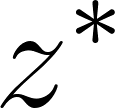
](https://www.codecogs.com/eqnedit.php?latex=z%5E%7B*%7D#0)). The Poisson-gamma mixture is specified as:

[
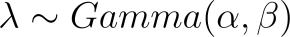
](https://www.codecogs.com/eqnedit.php?latex=%5Clambda%20%5Csim%20Gamma(%5Calpha%20%2C%5Cbeta%20)#0),

[
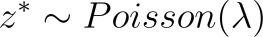
](https://www.codecogs.com/eqnedit.php?latex=z%5E%7B*%7D%5Csim%20Poisson(%5Clambda%20)#0),

where [
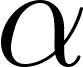
](https://www.codecogs.com/eqnedit.php?latex=%5Calpha#0) is the shape parameter and [
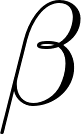
](https://www.codecogs.com/eqnedit.php?latex=%5Cbeta#0) is the rate parameter of the gamma distribution. We set [
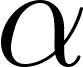
](https://www.codecogs.com/eqnedit.php?latex=%5Calpha#0) to the average of the [
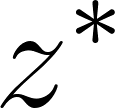
](https://www.codecogs.com/eqnedit.php?latex=z%5E%7B*%7D#0) in one CUS and [
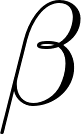
](https://www.codecogs.com/eqnedit.php?latex=%5Cbeta#0) to 1. We apply the ‘MCpoissongamma()’ function in the R package MCMCpack and simulate the mean value 1,000 times for downstream T-test for CUS value calculation.

- 1. **Calculating the posterior mean of cellular baseline in each CUS of a cell**

The smoothed observed counts are first standardized to [0,1] using min-max normalization, as:

[
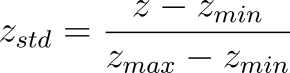
](https://www.codecogs.com/eqnedit.php?latex=z_%7Bstd%7D%20%3D%20%5Cfrac%7Bz-z_%7Bmin%7D%7D%7Bz_%7Bmax%7D-z_%7Bmin%7D%7D#0)

The prior of cellular baseline is set to the first quantile of the standardized distribution of the counts. Similar to the posterior calculation in mean of gene expression, the posterior mean of the cellular baseline in each CUS is generated by Poisson-gamma mixture for 1,000 simulations.

- 1. **Calculating the CUS value**

T-test is used to determine the statistical significance (denoted as [
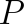
](https://www.codecogs.com/eqnedit.php?latex=P#0)) between the posterior mean of gene expression and the posterior mean of cellular baseline. Note that both of these mean values are simulated 1,000 times, ensuring the stability of the statistical test. The CUS value is finally calculated as:

[
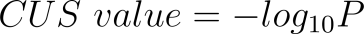
](https://www.codecogs.com/eqnedit.php?latex=CUS%5C%20value%20%3D%20-log_%7B10%7DP#0)

1. **Hierarchical artificial intelligence (AI) models**

**2.1 Model 1: CTC identification**

Model 1 was constructed based on an attention mechanism for identifying CTCs. In the input layer, a continuous linear projection transforms 553 CUS features of each cell into embedding vectors of dimension 8. These embeddings are processed through an encoder layer with 4 attention heads and a hidden dimension of 3, enhanced by ReLU activation to strengthen nonlinear representation capabilities. To incorporate biological context, a sinusoidal function is employed to encode the cell type associated with each CUS feature (e.g., 1 for tumor cells, 0 for other cells) into pseudo-positional vectors. The output layer utilizes a cross-entropy loss to classify cells as either tumor cells or non-tumor cells.

**2.2 Model 2: Tracing the tumor tissue of origin**

Model 2, designed to trace the tumor tissue of origin, follows a structure similar to that of Model 1 but incorporates several key modifications. The input dimension of 3356 is first transformed through a 256-dimensional hidden layer into 180 dimensions, which are then projected into 32-dimensional vectors. These features are processed through an encoder layer with 2 attention heads and a hidden dimension of 3, enhanced by a LeakyReLU activation function with a negative slope of 0.2 to strengthen nonlinear representation capabilities. The model employs a categorical cross-entropy loss to optimize performance in multi-class classification. The schematic of the models’ architecture is presented in Figure S1C.

**2.3 The training of the AI models**

Data partitioning was performed as follows:

- Training and validation set: 90% of the patient samples (Tables S1–S2) were used for training and validation, with a 9:1 split ratio;
- Independent test set: The remaining 10% (Tables S1–S2) were employed for performance evaluation of Model 1 and Model 2 of panCTC, as well as for comparative analysis between the proposed panCTC method and existing methods.

The hyperparameter configuration during training is as follows:

- Optimizer: Adam with an initial learning rate of 1e–3 and a weight decay coefficient of 1e–3;
- Batch size: 256 (dynamically adjusted based on GPU memory);
- Training epochs: 100, coupled with an early stopping mechanism (patience set to 5 epochs);
- Dropout rate: 0.05 for Model 1 and 0.2 for Model 2;
- Learning rate scheduling: The learning rate is reduced by a factor of 0.5 with a patience of 5 epochs until it reaches 1e-6.

**2.4 The evaluation metrics for the AI models**

To address the inherent class imbalance in PBMC data where circulating tumor cells (CTCs) are extremely rare, we employed a comprehensive set of evaluation metrics specifically designed to assess the performance of our AI models in both binary CTC classification and multi-class tissue origin identification.

For the critical task of binary CTC identification, we evaluated model performance using sensitivity, specificity, and a spectrum of F-scores (F₀.₅, F₁, and F₂). This multi-faceted approach allows for differential weighting between precision and recall: F₀.₅ places greater emphasis on precision to minimize false positives, F₂ prioritizes recall to reduce missed CTC detections, and F₁ maintains an equal balance between these two competing objectives.

For the multi-class classification task of tracing tumor tissue origins across twelve candidate primary tumor types, we reported overall accuracy while using the random prediction baseline (1/12 ≈ 0.083) as a critical reference point for interpreting model performance. This dual evaluation framework ensures rigorous assessment of both the detection capability and tissue-tracing precision of our panCTC system.

**Supplementary Figures**

**Figure S1 Overview of the panCTC framework for CTCs detection from scRNA-seq data of PBMCs**. (A) Workflow of calculating CUS values at single-cell resolution. Genes are aligned by genomic coordinates and their expression values are smoothed using polynomial dynamic linear modeling. The CUS value quantifies transcriptional activity within a chromosomal region (default: 10 consecutive genes) by comparing observed expression against a cell-specific baseline (defined as the first quantile of expression). Posterior distributions of gene sets are estimated via Markov chain Monte Carlo, and statistical significance between distributions is assessed with a t-tests. The CUS value is defined as -log_10_(*p*). (B) Architecture of Model 1 in panCTC, an attention-based neural network that distinguishes CTCs from other blood cells. (C) Architecture of Model 2, a similar attention-based neural network that classifies CTCs into one of twelve primary tumor types. Both models employ fully-connected layers and a transformer-style attention mechanism to capture long-range dependencies among CUS features.

**Figure S2 Correlation between scATAC-seq chromatin accessibility and CUS values across cell types**. (A) Chromatin accessibility peaks (at left) at marker-gene loci are, to some extent, associated with CUS values (at right) for representative markers: *CLU* (cancer cells), *FCN1* (monocytes), *CD3D* (T cells), *NACM1* (NK cells), *CD79A* (B cells), and *CD1C* (DCs). (B) Similar associations are observed in two subclusters of kidney cancer cells, using marker genes *KCNT2*, *GRM5*, *CLU*, *KRT19*, *KRT8*, and *KRT18*. (C) Genes, including transcription factors, involved in tumor proliferation, cell cycle, and apoptosis, which located in CUS regions highly correlated with ATAC-seq peaks (> 0.7).

scATAC-seq data are from 10x Genomics (10k_Human_PBMC_Multiome_v1.0 and Human_Kidney_Cancer_Nuclei, obtained from https://www.10xgenomics.com/). CUS values are derived from scRNA-seq data and linearly normalized to [0, 1].

**Figure S3 CUS values capture cell-type-specific transcriptional signatures.** (A) CUS regions containing classical marker genes exhibit the highest values in the corresponding cell type. For instance, the immune cell marker *PTPRC* (*CD45*) was located in state C255, where immune cells exhibited significantly higher CUS values than cancer cells. Furthermore, the CUS values associated with marker genes in cancer cells (*EPCAM*), monocytes (*FCN1*), T cells (*CD3D*, *CD8A*), NK cells (*NCAM1*), B cells (*CD79A*, *HLA-DRA*), and DCs (*CD1C*) all had the highest values in their respective cell types. For each CUS, t-test analysis is performed for different cell types, with random samples of n = 100 cells per cell type. (B) Gene‑set enrichment analysis of immune‑specific CUS regions reveals significant enrichment of immune‑related pathways (top), while tumor‑specific CUS regions are enriched for cancer‑associated pathways (bottom).

**Figure S4 CUS values enable cross-platform integration while preserving cell-type signatures**. (A) Raw UMI counts (upper) vary substantially across scRNA-seq platforms (10x Genomics, Smart-seq, and DNBelab-C4). However, CUS values converted by panCTC (lower) are at the same level and comparable. (B) The CUS values show superior performance in terms of platform integration and cell type clustering, comparable to the gene expression levels after Seurat treatment, indicating that the CUSs are capable of capturing certain inherent cellular biological signatures that overcome the barrier of technical variations. (C) Spearman correlation between CUS values and gene counts in T cells is moderate (median ρ = 0.535), confirming that CUS is not a simple reflection of transcript abundance (Kruskal-Wallis test)

**Figure S5 Performance evaluation of panCTC Model 1 for CTC identification**. (A) Validation was performed using pseudo-PBMC datasets constructed by mixing immune cells (2,720–10,000 cells) with low numbers of tumor cells (5–500 cells). Immune cells were derived from two sources: (i) simulated PBMCs with typical immune proportions, and (ii) real PBMCs from healthy donors (GSE199994, GSE155698). Tumor cells included published CTCs (single/clusters), primary cancer cells, and metastatic cancer cells, none of which were used during training. (B–D) panCTC achieved high performance for: (B) published CTCs (6 to 113 cells), (C) published primary cancer cells (5 to 500 cells), and (D) published metastatic cancer cells (5 to 500 cells). Mean (± SD) metrics across pseudo-PBMC tests included: sensitivity (0.962 ± 0.079), specificity (1.0 ± 0), precision (1.0 ± 0), and F-score (F_0.5: 0.996 ± 0.014, F_1: 0.990 ± 0.032, F_2: 0.985 ± 0.047). (E) panCTC yielded the lowest false-positive rate (0.001 ± 0.001) on healthy-donor PBMCs (n = 21). It outperformed other methods: 0.006 ± 0.02 for scATOMIC; 0.011 ± 0.026 for iCTC; 0.112 ± 0.178 for CopyKat; 0.67 ± 0.249 for CTCTracer; 0.226 ± 0.091 for scGPT; 0.252 ± 0.058 for scFoundation. (F-H) Head-to-head comparisons of sensitivity and specificity of panCTC and six state-of-the-art methods on the 102 unseen pseudo-PBMC datasets for (F) published CTCs (n = 15 datasets), (G) primary cancer cells (n = 72 datasets), and (H) metastatic cancer cells (n = 9 datasets). PanCTC (sensitivity: 0.987 ± 0.047 and specificity: 0.999 ± 0.002) outperformed all other methods on unseen 102 pseudo-PBMC datasets: 0.674 ± 0.399 and 0.999 ± 0.001 for scATOMIC; 0.731 ± 0.434 and 0.280 ± 0.322 for CTCTracer; 0.792 ± 0.308 and 0.985 ± 0.061 for scGPT; 0.818 ± 0.318 and 0.921 ± 0.137 for scFoundation; 0.279 ± 0.391 and 0.841 ± 0.161 for CopyKAT; 0.053 ± 0.210 and 0.957 ± 0.170 for iCTC.

**Figure S6 Performance evaluation of panCTC Model 2 for tracing the tissue origin of CTCs.** (A) Validation was performed using publicly available and newly sequenced primary cancer cells, metastatic cancer cells, isolated and validated CTCs which were previously published, and CTCs identified from cancer patient PBMCs with known cancer types. (B–F) Top‑1 and top‑3 classification accuracy across different sample categories: (B) primary cancer cells (n = 8 to 9 samples), (C) metastatic cancer cells (n = 8 samples), (D) published single CTCs (n = 12 datasets), CTC clusters (n = 3 datasets) and newly sequenced CTCs (n = 2 datasets), (E) CTCs identified by panCTC in PBMCs from patients with matched tumor scRNA-seq data (n = 3 to 17 datasets), and (F) CTCs identified by panCTC in PBMCs from patients with only known cancer types without scRNA data of matched tumors (n = 1 to 7 datasets). PanCTC achieved exceptional performance: top-1 accuracy 0.999 ± 0.003 and top-3 accuracy 1 ± 0.002 for primary cancer cells; 0.739 ± 0.149 and 0.923 ± 0.067 for metastatic cancer cells; 0.330 ± 0.291 and 0.529 ± 0.335 for experimentally isolated and validated CTCs. (g-i) Comparison of panCTC with current methods such as scATOMIC, CTCTracer, scFoundation, and scGPT in classifying cancer types of: (G) published single CTCs or CTC clusters (6 to 113 cells), (H) published primary cancer cells (3 to 100 cells), and (I) published metastatic cancer cells (3 to 100 cells). PanCTC achieved highest top-1 accuracy in all comparisons: 0.995 ± 0.025 for primary cancer cells; 0.660 ± 0.258 for metastatic cancer cells; and 0.475 ± 0.253 for experimentally isolated and validated CTCs. The dashed line indicates random prediction accuracy (0.083 for 12 cancer types). Statistical significance was determined by two-tailed t-test.

**Figure S7 Subclustering of PDAC primary cancer cells and CNV similarity between CTCs and primary subclusters.** (A) UMAP of malignant cells from a PDAC sample (GSE155698) clustered at resolution 0.1. (B) Number of cancer cells per subcluster. (C) CNV profiles of CTCs identified from PBMCs show clear similarity to CNV patterns of matched primary-tumor subclusters.

**Figure S8 Subclustering of newly sequenced colorectal cancer (CC) samples and CNV similarity between CTCs and primary subclusters.** (A) UMAP of malignant cells from each CC sample clustered at resolution 0.5. (B) Number of cancer cells per subcluster in each CC sample. (C) CNV profiles of CTCs from PBMC sample P2Dur share several gain/loss events with subclusters of matched primary cancer cells (T2Dur). (D) CNV profiles of CTCs from PBMC sample PreP3 (patient CC Pair 3) share several gain/loss events with subclusters of matched primary cancer cells (PreT3).

**Figure S9 Somatic mutations shared between newly sequenced CTCs and matched primary cancer cells**. CTC-primary pairs were derived from a breast cancer patient (6 CTCs + 6 primary cancer cells) and a colorectal cancer patient (17 CTCs + 7 primary cancer cells) and sequenced on the Smart-seq platform. All cells were correctly classified as cancer cells by panCTC (accuracy = 1.0). Several cancer‑associated mutations (*CDKN2C, LMNA, MUC1, PDE4DIP, HRAS, B2M, COL1A1,* and *PIK3CA*) were detected in both CTCs and primary cells from both patients.

**Figure S10** **Biological characteristics of panCTC-identified CTCs are consistent with established CTC knowledge**. (A) Heatmap of differentially expressed genes between CTCs and paired immune cells in PBMC datasets from ten cancer types, including cervical cancer (CC), non-small cell lung cancer (NSCLC), colorectal cancer (CRC), Pancreatic ductal adenocarcinoma (PDAC), nasopharyngeal carcinoma (NPC), ovarian cancer (OVC), breast cancer (BC), gastric cancer (GC), hepatocellular carcinoma (HCC), and melanoma. CTCs express known markers (e.g., *EPCAM*, *KRT18*, *KRT8*, *KRT7*, *SOX9,* and *SOX4*). PDAC CTCs also exhibited high expression levels of *SPINK1*, which is associated with pancreatic function, as well as *MUC1*, *CEACAM6*, *ERBB3*, and *CDH1*, which are associated with cell proliferation, invasion, and metastasis. Significant markers are denoted with asterisks (*, *p* < 0.05; **, *p* < 0.01; ***, *p* < 0.001). (B) EMT scores of CTCs, calculated using the Kolmogorov-Smirnov method, from each cancer type, compared with T cells (10x Genomics pbmc_10k_v3), normal fibroblasts (GSE163973), and epithelial cells (GSE168652) (n = 200 cells per cell type). CTCs from PDAC and BC patients were more likely to exhibit both mesenchymal and epithelial tendencies, while CTCs from other cancer types were primarily mesenchymal. (C) Gene-set enrichment analysis on CTCs reveals significant enrichment of pathways associated with cell junctions, adhesion and migration, platelet function, and metabolism.

**Figure S11 Transcriptional profiles and pathway enrichment of *EPCAM*^+^ vs. *EPCAM*^–^ CTC-like cells.** (A) Differentially expressed genes between *EPCAM*^+^ CTCs and *EPCAM*^–^ CTC-like cells. (B) Gene-set enrichment for *EPCAM*^+^ CTCs highlights pathways related to cytoskeleton organization and cell adhesion. (C) Gene-set enrichment for *EPCAM*^–^ CTC-like cells highlights pathways related to oncological pathways, immune functions, and decreased metabolism

**Figure S12 Cell‒cell interactions between CTCs and immune cells in PBMCs**. (A) Tumor-specific ligand-receptor pairs predominantly expressed from CTCs to immune cells. (B) Tumor-specific ligand-receptor pairs predominantly expressed from immune cells to CTCs. For instance, the unique presence of CD44-collagen interactions in the PBMCs of PDAC patients indicates the extravasation potential of PDAC CTCs traversing the vasculature. (C) Common ligand-receptor pairs predominantly expressed from CTCs to immune cells across various cancer types. For instances, ITGB-ICAM interactions with T/NK/megakaryocyte/DC cells, suggesting potential roles for transendothelial migration and cell adhesion in CTC cluster formation; major histocompatibility complex class I (MHC I) interactions with T/NK/MKI67+ cells, indicating a possible strategy immune-evasion mechanism via platelet-derived pseudonormal phenotype acquisition; CLEC-KLRB1 interactions, likely protecting CTCs from NK cell-mediated lysis. (D) Common ligand-receptor pairs predominantly expressed from immune cells to CTCs. For instances, LGALS9-CD44, which may enhance immunosuppressive functions in mesenchymal CTCs; CD99-CD99 and MIF-CD74, implicated in endothelial basement membrane penetration and extravasation. The top color strips indicate PBMC datasets used for predicting CTCs; the left color strips categorize ligand-receptor groups.

**Figure S13 Clinical relevance of *in situ*-identified CTCs.** In total, three PBMC scRNA datasets with clinical cancer stage information are available (Table S8). (A) Patients are classified into two groups: those with metastasis (either invading nearby lymph nodes or colonizing new sites) and those without metastasis. Identified CTCs are also grouped according to metastasis status. (B) These CTCs are catagorized into *EPCAM*^+^ CTC and *EPCAM*^–^ CTC-like cell subpopulations. (C) In these datasets across three cancer types, all *EPCAM*^+^ CTCs are observed in metastatic breast cancer patients. However, the presence of *EPCAM*^–^ CTC-like cells is observed in all patients, regardless of the patients’ metastasis status. (D) For *EPCAM*^–^ CTC-like cells, the upregulated genes of CTCs with metastasis (red dots) and CTCs without metastasis (blue dots) are shown in the MA plots. (E) For *EPCAM*^–^ CTC-like cells, the pathways enriched in CTCs with and without metastasis are also shown. The genes with higher expression levels and the pathways enriched in EPCAM^–^ CTC-like cells are associated with the metastasis status of cancer patients. To detect target genes of CTCs involved in cancer metastasis, a new dataset of ovarian cancer (PRJCA005422) is generated with paired scRNA data from PBMCs, metastatic tumors, lymph nodes, and ascites samples. CTCs are identified from PBMCs using panCTC. (F) Ligand-receptors from CTCs to immune cells that are mainly exhibited in *EPCAM*^–^ CTC-like cells, rarely found in *EPCAM*^+^ CTCs. (G) Survival analyses of partial marker genes of ovarian cancer patients (PRJCA005422) from both the TCGA and other GEO databases. Survival curves were compared using the log-rank test. These marker genes associated CTCs identified by panCTC were significantly correlated with poor overall survival in ovarian cancer patients.
